# The control of acidity in tumor cells: a biophysical model

**DOI:** 10.1038/s41598-020-70396-1

**Published:** 2020-08-12

**Authors:** Nicola Piasentin, Edoardo Milotti, Roberto Chignola

**Affiliations:** 1 Department of Physics, University of Trieste, Via Valerio 2, 34127 Trieste, Italy; 2 Department of Biotechnology, University of Verona, Strada Le Grazie 15 - CV1, 37134 Verona, Italy; 3 Present Address: Department of Chemical and Process Engineering, University of Surrey, Guildford, GU2 7XH UK; 4 Present Address: Unilever Research Colworth, Colworth Park, Sharnbrook, Bedfordshire, MK44 1LQ UK

**Keywords:** Biomedical engineering, Biological physics

## Abstract

Acidosis of the tumor microenvironment leads to cancer invasion, progression and resistance to therapies. We present a biophysical model that describes how tumor cells regulate intracellular and extracellular acidity while they grow in a microenvironment characterized by increasing acidity and hypoxia. The model takes into account the dynamic interplay between glucose and $\hbox {O}_2$ consumption with lactate and $\hbox {CO}_2$ production and connects these processes to $\hbox {H}^+$ and $\hbox {HCO}_3^-$ fluxes inside and outside cells. We have validated the model with independent experimental data and used it to investigate how and to which extent tumor cells can survive in adverse micro-environments characterized by acidity and hypoxia. The simulations show a dominance of the $\hbox {H}^+$ exchanges in well-oxygenated regions, and of $\hbox {HCO}_3^-$ exchanges in the inner hypoxic regions where tumor cells are known to acquire malignant phenotypes. The model also includes the activity of the enzyme Carbonic Anhydrase 9 (CA9), a known marker of tumor aggressiveness, and the simulations demonstrate that CA9 acts as a nonlinear $\hbox {pH}_i$ equalizer at any $\hbox {O}_2$ level in cells that grow in acidic extracellular environments.

## Introduction

Acid homeostasis in animal tissues is achieved by active dynamic processes. In physiological conditions, the pH of tissues is maintained between 7.35 and 7.45 in spite of constant metabolic acid production by cells. At the microscopic level, cells must finely regulate their own internal pH to around 7.2 to avoid death^1–3^. Cellular acid homeostasis is carried out by active transport of acid/base equivalents across the cell membranes into the extracellular spaces.

Dysregulation of pH is a well-known hallmark of solid tumors^1–3^. The tissue of solid tumors is characterized by the presence of an irregular network of blood vessels, causing a spatially heterogeneous delivery of nutrients such as glucose and oxygen to tumor cells^1–4^. As the consequence, the inner regions of solid cancers that are distant from blood vessels become hypoxic and acidic. Cancer cells adapt to such adverse environments through a series of molecular changes that involve an increased expression of nutrient and ion transporters and enzymes (reviewed in^1,3,5^). For example, hypoxia activates the Hypoxia Inducible Factor-1$\alpha$ (HIF-1$\alpha$) that up-regulates the transcription of glucose transporters and of enzymes involved in glucose metabolism. Because of hypoxia, glucose is converted mainly to lactic acid through the glycolytic pathway to produce energy under the form of ATP, and the increased production of lactate reduces the pH of the extracellular spaces. A drop in intracellular pH, in turn, increases the activity of lactate and of various ion transporters that collectively contribute to recover intracellular acid homeostasis^1,3,5^. Hypoxia also causes the increased expression of some membrane-bound enzymes such as Carbonic Anhydrase (CA) that, on the cell surface, catalyzes the hydration of carbon dioxide ($\hbox {CO}_2$) to protons ($\hbox {H}^+$) and bicarbonate ($\hbox {HCO}_3^-$) ions. While the $\hbox {H}^+$ ions contribute to the acidity of the extracellular milieu, $\hbox {HCO}_3^-$ ions can be transported back into the cells and increase the buffering potential of the intracellular environment^1,3,5^, further contributing to maintain the intracellular pH at normal values.

It has recently been pointed out^1,3^ that changes in the control of intracellular and extracellular acidity in the tissue of solid tumors are associated with many phenotypic changes of cancer cells with important implications in tumorigenesis, cancer progression, cancer diffusion, escape from immune surveillance and resistance to therapies. For example, microscopic examination of the tumor/normal tissue interface shows that peritumoral acidity drives tumor invasion in the surrounding normal tissue, with the regions of highest tumor invasion corresponding to those of lowest pH. In these regions the environmental pH reaches values that are toxic for normal but not for tumor cells^2^.

Biophysical models can help to disentangle the intricate relationships between regulatory biochemical networks and give support to the interpretation of experimental evidence which is rapidly accumulating in this field. In this paper we describe a comprehensive biophysical model of the control of acidity in tumor cells. We study the action of key molecular actors in acid homeostasis of cancer cells, and investigate to which extent hypoxia and environmental acidosis influence their behavior. We focus on the dynamic interplay between lactate, proton, bicarbonate transporters and CA enzyme, and their regulation by oxygen and both extracellular and intracellular pH. The model includes the bicarbonate buffer that acts both in the extracellular and intracellular milieux and it incorporates results from our previous modeling efforts concerning tumor cell metabolism^6–8^. In particular, our previous models provide values for the rates of glucose and oxygen uptake, lactate and $\hbox {CO}_2$ production and lactate/$\hbox {H}^+$ transport across cell membranes through specific transporters that have already been validated with experimental data. Finally, we fix the model parameters by combining information from a number of experiments carried out with different tumor cell systems.

## Results

### Preliminary considerations, model assumptions and parameters

We start from the rather detailed model of tumor cell metabolism and growth that we developed in our previous research^6–8^ which successfully reproduces the observed behavior of tumor cells in both liquid (e.g. blood tumors) and solid tumors. In particular, for the current work we have excerpted from that model the part that describes the rates of glucose conversion to lactic acid and oxygen consumption. We remark that the model in^6–8^ has been set up with the minimal set of chemical and biochemical pathways that drive the dynamics of metabolism and that are common to most, if not all, tumor cells.

**Figure 1:**
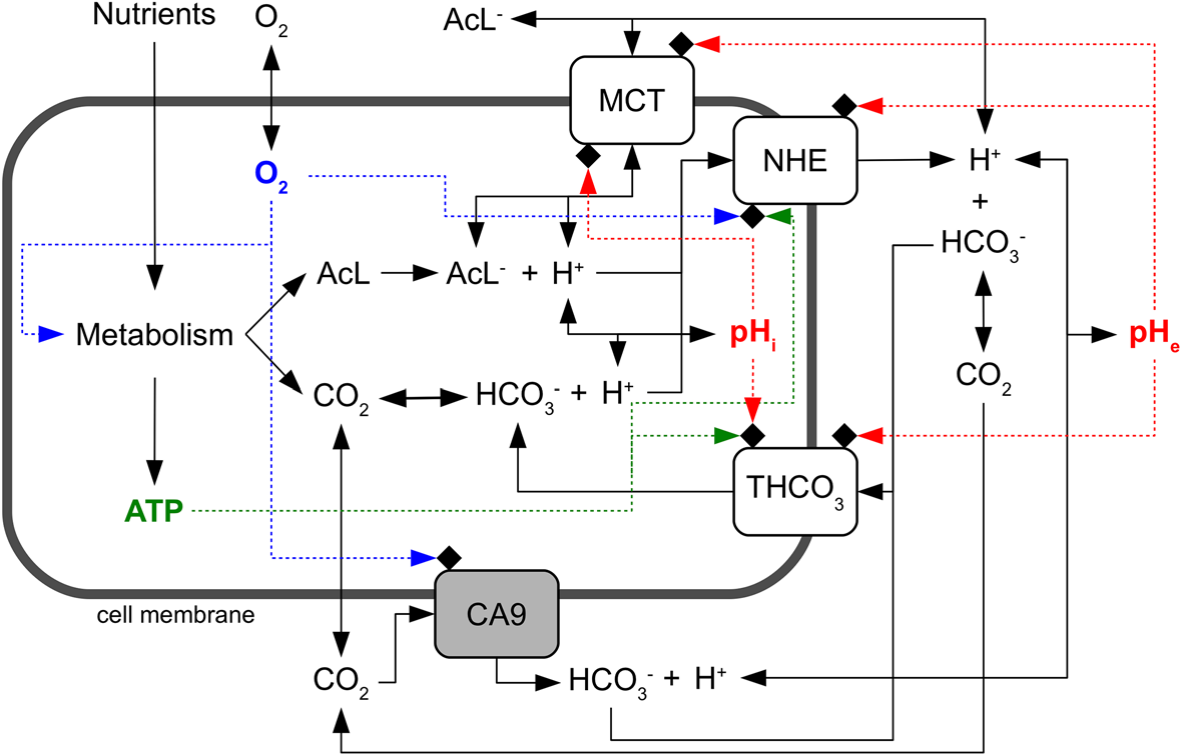
Layout of the model of acidity control in tumor cells. A cell takes up from the environment nutrients and oxygen which are then converted by cell metabolism to lactic acid, $\hbox {CO}_2$ and ATP. Lactic acid dissociates to lactate and $\hbox {H}^+$ ions, whereas $\hbox {CO}_2$ reversibly hydrates to $\hbox {HCO}_3^-$ and $\hbox {H}^+$. These chemical species diffuse through cell membranes ($\hbox {CO}_2$) or are actively transported outside and eventually inside the cell by means of specific protein transporters. We consider monocarboxylate (MCT), sodium-hydrogen exchanger (NHE) and generic bicarbonate ($\hbox {THCO}_3$) transporters. We also model the activity of the membrane-bound enzyme Carbonic Anhydrase 9 (CA9). Chemical reactions are indicated by solid lines and the regulatory pathways by dashed lines. Proton concentrations inside and outside the cell are used to compute the intracellular ($\hbox {pH}_i$) and the extracellular ($\hbox {pH}_e$) pH. Detailed information on each pathway is given in the main text.

Unlike the metabolic model in^6–8^, here we must follow the dynamics of $\hbox {CO}_2$, $\hbox {HCO}_3^-$ and $\hbox {H}^+$, both inside and outside a tumor cell. The inputs of the model are the rates of lactate and $\hbox {CO}_2$ production (Fig. 1) that depend on how cells take up nutrients, such as glucose, and convert them to ATP through the glycolytic and the oxidative phosphorylation pathways. Lactic acid dissociates immediately to lactate and $\hbox {H}^+$ ions, and both ions are transported through the cell membrane by means of the bi-directional monocarboxylate transporters MCT^6–8^. We remark that this part of the model impacts the rate of change of both intracellular and extracellular pH (from now on $\hbox {pH}_i$ and $\hbox {pH}_e$, respectively), and oxygen is assumed to diffuse freely through the cell membrane and its consumption rate is used to determine the rate of $\hbox {CO}_2$ production.

Intracellular $\hbox {H}^+$ ions are transported outside the cell by means of unidirectional sodium-hydrogen exchangers NHE^1^. Different $\hbox {HCO}_3^-$ transporters on the other hand are known to drive the flux of bicarbonate ions through the cell membrane. Some of them import or export $\hbox {HCO}_3^-$ by exchanging $\hbox {Cl}^-$ anions and the transport may depend or not on the presence of $\hbox {Na}^+$ cations^1^. Experimental works, however, have shown that the efficiency of $\hbox {HCO}_3^-$ transport in different cell systems is quite similar, and that the import of $\hbox {HCO}_3^-$ is fundamental in tumor cells where it is dominated by the activity of the $\hbox {Na}^+$-dependent $\hbox {Cl}^-/\hbox {HCO}_3^-$ exchanger^9,10^. Therefore, we consider the import activity of a generic $\hbox {HCO}_3^-$ transporter ($\hbox {THCO}_3$ in Fig. 1) which, as a first approximation, assumes the average biochemical characteristics of the $\hbox {Na}^+$-dependent $\hbox {Cl}^-/\hbox {HCO}_3^-$ exchanger. We finally model the activity of the membrane-bound Carbonic Anhydrase 9 (CA9) enzyme that catalyzes, on the cell surface, the hydration of $\hbox {CO}_2$. This is an important path since CA9 has been found to be expressed by many solid tumors of different histotypes, and its activity has been correlated to tumor progression and growth^11–13^.

It should be noted in Fig. 1 that we do not take into account a possible effect of the extracellular pH on CA9 activity. Previous work has shown that CA9 in cell membrane extracts is sensitive to low pH and is completely inhibited at pH 6.0^14^, its pH sensitivity being much steeper than that of other CA isoforms^15^. This observation, however, is at odd with findings obtained using high-resolution techniques with purified enzyme: they showed that the catalytic domain of human CA9, but not of other isoforms, is stable and active still at very low non-physiologic pH but inactive at $\hbox {pH} > 8.0$^16^. Because of these discrepancies, and since we do not want to focus on some specific cell system but rather to keep the model as general as possible, we decided to leave off the possible pH sensitivity of CA9 from the present model. Our modelling strategy is flexible enough to incorporate additional specific details when available, provided they are based on firm experimental conclusions.

We model the kinetics of ion transporters, and of CA9 activity as well, with the Michaelis-Menten/Hill formalism that is described by the following general equation:

$$\begin{aligned} \frac{d[X]_{C,c}}{dt}=\frac{V_{\mathrm{max}}[X]_{C,c}^h}{K_\text {m}^h+[X]_{C,c}^h} \end{aligned}$$

where $[X]_{C,c}$ is the molar concentration of a given chemical species inside ($[X]_C$) or outside ($[X]_c$) the cell, $V_{max}$ and $K_m$ are the Michaelis-Menten parameters and *h* is the Hill exponent ($h>0$).

We assume that:

- $\hbox {CO}_2$ can freely diffuse through the cell membrane;
- $\hbox {CO}_2$ diffusion is driven by the concentration gradient across the membrane and its only important component is the one directed normally with respect to the cell membrane;
- the diffusion kinetics of charged ions through the cell membrane are much slower than the kinetics of the other processes in which they are involved, and thus the diffusion of charged ions is negligible;
- the mixing of all chemical species in the cell and in the external environment is instantaneous;
- within the short characteristic times of the considered chemical reactions the cell volume is constant.

In this work the variables take the following units for length, mass and time, respectively: $\upmu \hbox {m}$, pg and s. Molar concentrations (M) have always been converted to mass units by taking into account the volume of the cell ($\hbox {V}_C$, cell volume is computed by approximating a cell to a sphere of given radius $\hbox {r}_C$) or of the environment ($\hbox {V}_c$) and the molecular mass (MW) of chemical species.

The model defined by the set of differential equations 11 has several parameters. We extensively searched the scientific literature to find their values, and when these values were not directly available they were obtained by fit of specific equations to reported experimental data. Experimental evidence was also used to model regulatory functions given by Eqs. 3, 5, 7 and 10 that tune the activity of transporters and CA9 enzyme as the function of local pH, ATP and/or oxygen availability. The full strategy is detailed in the Supplementary Material and all parameter values are listed in Table 1.

**Table 1:** Values of model parameters

Parameter	Value	Unit	Reference
$\hbox {MW}_{\mathrm{H}}$	1	$\hbox {g mol}^{-1}$	–
$\hbox {MW}_{\mathrm{CO}_2}$	44	$\hbox {g mol}^{-1}$	–
$\hbox {MW}_{\mathrm{O}_2}$	32	$\hbox {g mol}^{-1}$	–
$\hbox {MW}_{\mathrm{HCO}_3}$	61	$\hbox {g mol}^{-1}$	–
$\hbox {MW}_{\mathrm{AcL}}^{\mathrm{a}}$	90.1	$\hbox {g mol}^{-1}$	–
$\hbox {P}_{\mathrm{M,CO}_2}^{\mathrm{b}}$	$3.2\times 10^4$	$\upmu \hbox {m s}^{-1}$	^17^
gAcL	$3.8\times 10^{-4}$	$\hbox {pg s}^{-1}$	^6^
$\hbox {qO}_2$	$3.5\times 10^{-5}$	$\hbox {pg s}^{-1}$	^6^
$\hbox {k}_1$	0.144	$\hbox {s}^{-1}$	^18^
$\hbox {k}_2$	$1.9\times 10^5$	$\hbox {M}^{-1}\,\hbox {s}^{-1}$	^18^
$\hbox {V}_\text {maxAcL}$	$9.58\times 10^{-5}$	$\hbox {pg s}^{-1}\,\upmu \hbox {m}^{-2}$	^8^
$\hbox {K}_\text {mAcL}$	$0.405\times 10^{-3}$	$\hbox {pg}\,\upmu \hbox {m}^{-3}$	^8^
$\hbox {a2c}_\text {H}$_slope	1.5	–	^8^
$\hbox {a2c}_\text {H}$_thr	7	–	^8^
$\hbox {c2a}_\text {H}$_slope	1.5	–	^8^
$\hbox {c2a}_\text {H}$_thr	7	–	^8^
$\hbox {V}_\text {maxNHE}$	$5.15\times 10^{-7}$	$\hbox {pg s}^{-1}\,\upmu \hbox {m}^{-2}$	Fit of data in^9^
$\hbox {K}_\text {mNHE}$	$0.196\times 10^{-6}$	M	Fit of data in^9^
h	2.67	–	Fit of data in^9^
$\lambda _\text {NHE}$	0.076	–	Fit of data in^9^
$\hbox {pH}_\text {0,NHE}$	7.1	–	Fit of data in^9^
$\hbox {V}_\text {maxTHCO3}$	$2.02\times 10^{-5}$	$\hbox {pg s}^{-1}\,\upmu \hbox {m}^{-2}$	Fit of data in^19^
$\hbox {K}_\text {mTHCO3}$	$7.38\times 10^{-3}$	M	Fit of data in^19^
$\lambda _\text {THCO3}$	1.63	–	Fit of data in^9^
$\hbox {pHe}_\text {0,THCO3}$	6.85	–	Fit of data in^9^
$\gamma _\text {THCO3}$	4.2	–	Fit of data in^10^
$\hbox {pHi}_\text {0,THCO3}$	6.90	–	Fit of data in^10^
$\hbox {V}_\text {maxCA9}$	$9.47\times 10^{-2}$	$\hbox {pg s}^{-1}\,\upmu \hbox {m}^{-2}$	^20^
$\hbox {K}_\text {mCA9}$	$7.2\times 10^{-3}$	M	^21^
$\delta _\text {CA9}$	7.3	–	Fit of data in^22^

$^{\mathrm{a}}$ AcL=lactic acid/lactate.

$^{\mathrm{b}}$ Parameter values have been determined and fixed as described in the Supplementary Material section

Once determined, parameter values were fixed and no further tuned to adapt model outputs to data. This means that the model has no free parameters and is strictly predictive. As explained in the next section, for validation purposes we first used it to predict how the intracellular pH ($\hbox {pH}_i$) varies when cells are grown into environments with increasing acidity.

### Model validation with independent experimental data

Model validation was performed with independent experimental data, i.e. data that were not used to set parameter values. To this end we used the data in the paper by Song et al.^23^. In this paper Song et al. investigated the dependence of $\hbox {pH}_i$ on $\hbox {pH}_e$ in SCK cells (human choloangiocarcinoma cell line) in standard *in vitro* cultures. To the best of our knowledge no data concerning the direct expression of specific ion transporters and CA9 in these cells are available. However, the $\hbox {pH}_i$ of SCK cells was measured in experiments where cells were also treated with Amiloride and DIDS inhibitors. Amiloride inhibits $\hbox {Na}^+$ channels and thus inhibits sodium-hydrogen exchangers, whereas DIDS inhibits all bicarbonate-dependent transport mechanisms (see Song et al.^23^ and references cited therein). Thus, the expression of proton and bicarbonate transporters was functionally demonstrated in SCK cells. We do not know if SCK cells express CA9 but, as we shall see below (see Fig. 5), CA9 activity becomes negligible for $\hbox {pH}_i$ regulation when the extracellular volume becomes higher than $10^4$ cell volumes, i.e. when the extracellular volume exceeds $\sim 0.02\, \upmu \text {l}$ (the volume of 1 cell of radius $\sim 7\, \upmu \hbox {m}$ is $\sim 2\ \text {pl}$). The experiments were carried out with cells kept under standard culture conditions where the extracellular volume is much higher, and thus it is irrelevant whether SCK cells express CA9 or not. Data obtained with SCK cells can therefore be used to validate the core model as far as the regulation of $\hbox {pH}_i$ due to the activity of ion transporters is concerned.

The radius of SCK cells is not reported nor, to the best of our knowledge, it has been measured previously. This is important because our model equations take into account both cell volume (see Eqns. 1–11) and the cell surface (see e.g. $\hbox {CO}_2$ diffusion, Eq. 1) that are computed from cell radius under the assumption that cell geometry can be approximated by a sphere. Thus we run simulations for different cell radii whose values were taken within a reasonable range for animal cells.

**Figure 2:**
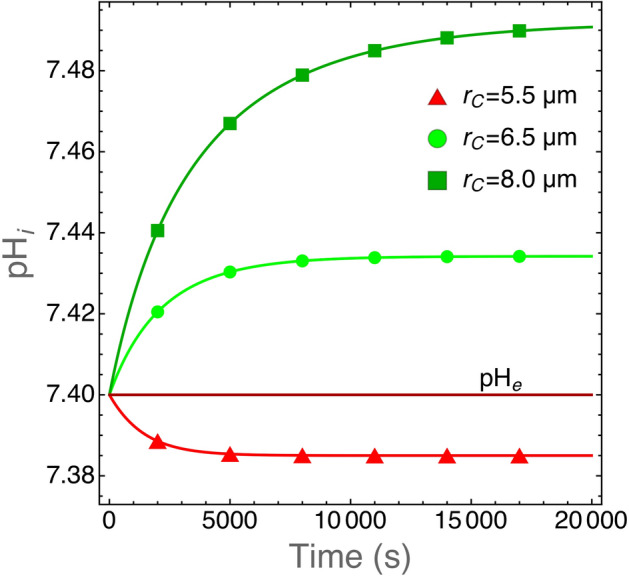
Plot of $\hbox {pH}_i$ as the function of time for cells with the indicated cell radii. We take $\hbox {r}_C$ values that are in the observed range for human tumor cells^24^. The model equations have been solved with the parameter values listed in Table 1. After an initial transient, $\hbox {pH}_i$ reach an equilibrium at physiological values and this shows that the model (and its numerical solution) is stable and provides quantitative results in good agreement with actual experimental observations. We also plot $\hbox {pH}_e$ for comparison. The extracellular pH does not vary because these runs were carried out for a limited time span and for cells growing in a large volume (1 mL) filled with fresh medium at physiological pH to mimic standard experimental conditions.

Figure 2 shows the model prediction for intracellular pH vs. cell size, under standard culture conditions. At equilibrium there is a difference of $\approx 0.1$ in pH between small and large cells ($\hbox {r}_C=5.5$ and $8.0\,\upmu \hbox {m}$, respectively, i.e. a volume ratio of $\simeq 3$) but $\hbox {pH}_i$ levels reach values that have actually been observed in tumor cells^23^. With the initial conditions discussed above, the simulations approach equilibrium quite fast and this indicates that the numerical solution of model equations is stable.

**Figure 3:**
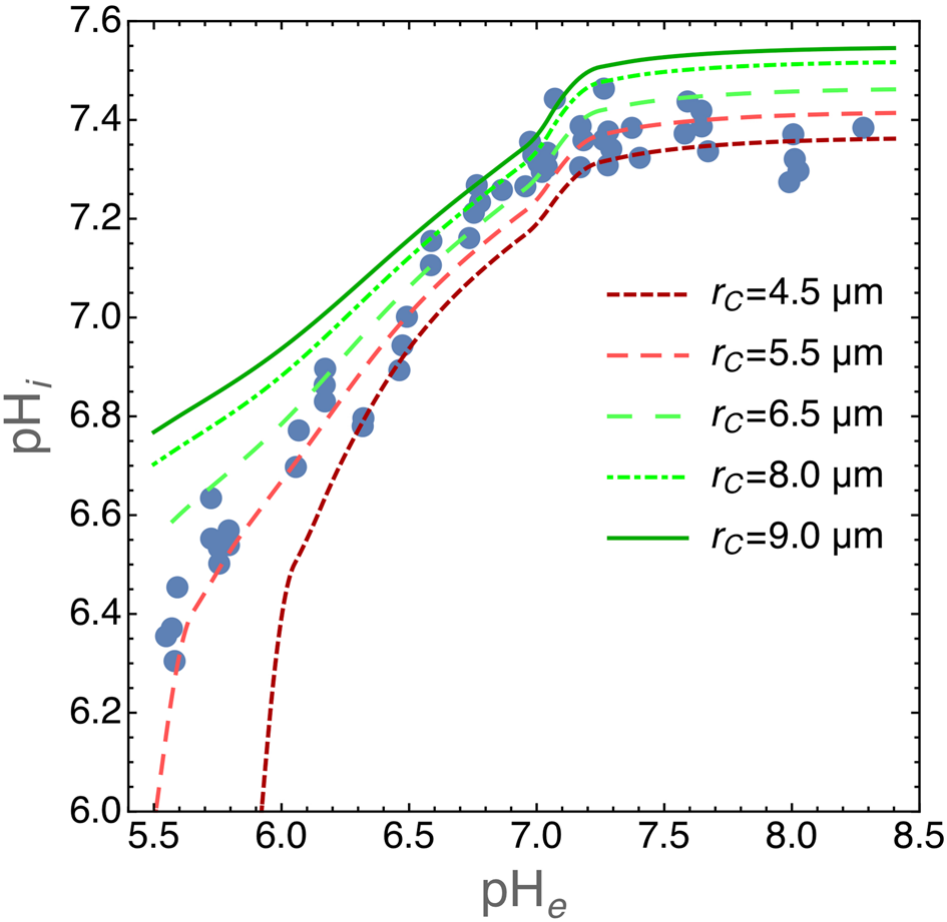
Plot of $\hbox {pH}_i$ for SCK cells grown in media with different $\hbox {pH}_e$ values. Experimental data have been redrawn from figure 2 in^23^ (closed circles). The lines show $\hbox {pH}_i$ values at equilibrium as predicted by our model for the indicated cell radii. It is important to note that these are not fits because our model does not have free parameters. Equilibrium was reached at $\Delta \text {pH}_i/\Delta t < 10^{-5}$. The volume of the environment was set at $\hbox {V}_c=10^{12}\, \upmu \text {m}^3 = 1 \text {ml}$.

The model predictions for $\hbox {pH}_i$ values in SCK cells grown in media with increasing acidity are shown in Fig. 3. We ran simulations with varying cell radius within a range of values which is reasonable for tumor cells, i.e. between 4.5 and 9 $\upmu \hbox {m}$^24^, and computed $\hbox {pH}_i$ at equilibrium. As shown in Fig. 2 the numerical solutions approach equilibrium with slower kinetics for increasing cell radii. We chose a conservative criterion to define the equilibrium condition and we halted the simulations when $\Delta \text {pH}_i/\Delta t < 10^{-5}$ was reached. In these simulations, the volume of the environment was set to $\hbox {V}_c=10^{12} \,\upmu \text {m}^3=1\,\text {ml}$, i.e. large enough to assure nearly constant $\hbox {pH}_e$ values throughout the simulation runs. Figure 3 shows that model predictions are in excellent agreement with the experimental data.

### Contribution of NHE and THCO3 transporters to $\hbox {pH}_i$ in normoxic or hypoxic environments

**Figure 4:**
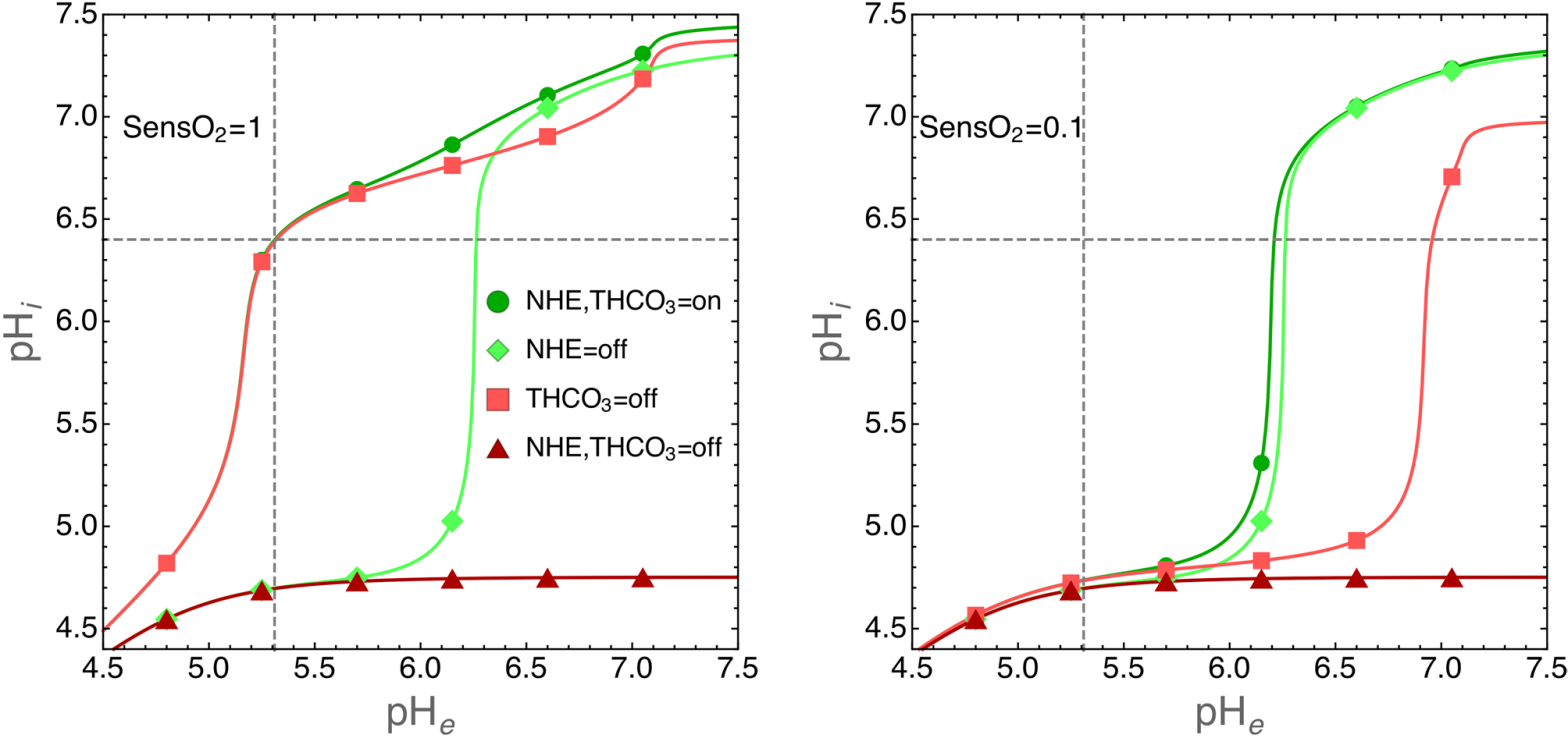
Contribution of NHE and THCO3 transporters to $\hbox {pH}_i$ in normoxic (left panel) or hypoxic (right panel) environments. Simulations were run with the following parameters: cells radius $\hbox {r}_C=6.5 \,\upmu \hbox {m}$ and environmental volume $\hbox {V}_c = 10^{12}\, \upmu \text {m}^3$. The intracellular pH was calculated at equilibrium (see also the legend to Fig. 3) as the function of the indicated $\hbox {pH}_e$ values. The activity of NHE and THCO3 transporters was switched off by setting the respective $\nu _{\mathrm{max}}$ parameters to 0. Environmental oxygen levels were tuned by setting the SensO2 parameter to 1 or to 0.1 (see the “Methods” section and the Supplementary Material for details). In both panels, dashed lines have been drawn to show the $\hbox {pH}_e$ value at which $\hbox {pH}_i=6.4$, a value largely compatible with cell life (see also the experimental data in Fig. 3 for a comparison).

We have used the model to study the biochemical mechanisms that allow tumor cells to survive to adverse environments. We have investigated the role of NHE and THCO3 transporters in the control of intracellular acidity by tumor cells exposed to normoxic or hypoxic environments. We ran several simulations by alternatively switching off the activity of NHE and THCO3 transporters, i.e. by setting the respective $\nu _{\mathrm{max}}$ parameters to 0. The results are shown in Fig. 4 where we plot the $\hbox {pH}_i$ values at equilibrium (see the previous section) as the function of environmental pH for cells grown under standard oxygen level or at 0.1 fraction thereof.

The simulations clearly show that under normoxic condition the contribution of the THCO3 transporter to $\hbox {pH}_i$ is negligible. Under this condition $\hbox {pH}_i$ is maintained to physiological levels thanks to the activity of NHE transporter that export $\hbox {H}^+$ ions outside the cells. On the contrary, THCO3 activity dominates in hypoxic environments.

### Role of Carbonic Anhydrase 9

**Figure 5:**
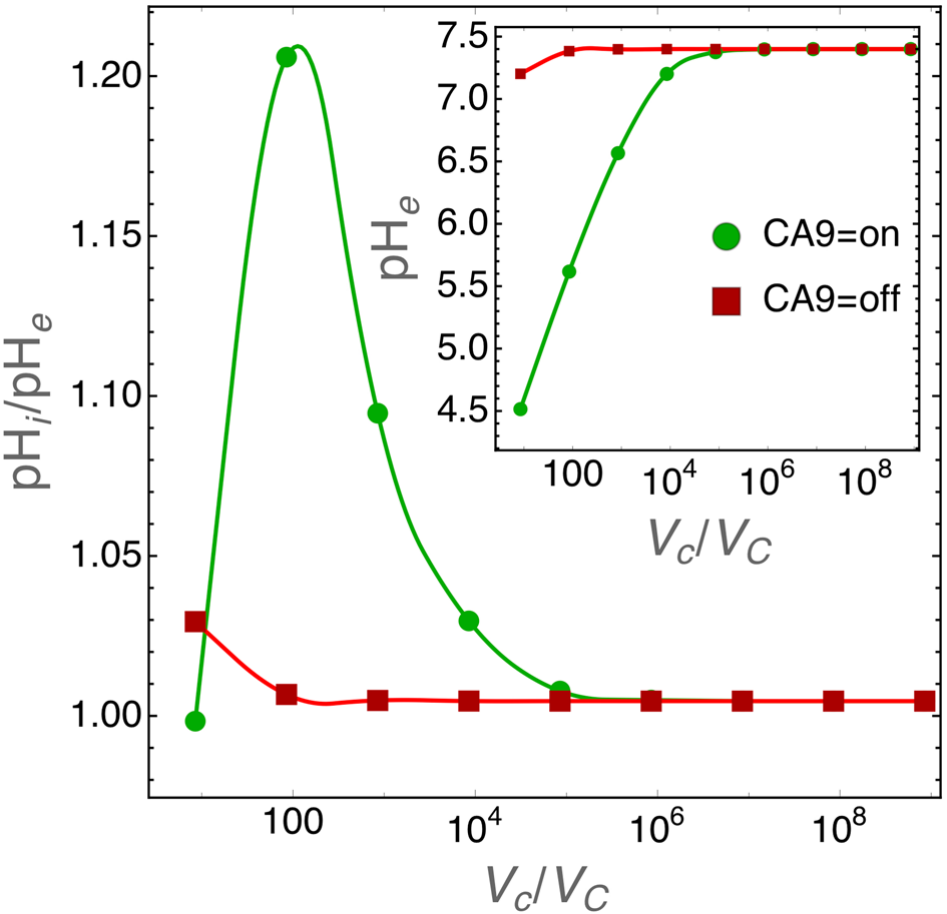
$\hbox {pH}_i$ regulation by CA9 for decreasing size of the extracellular volume. Cell radius was set to the average size of 6.5 $\upmu \hbox {m}$. The inset shows $\hbox {pH}_e$ values and the main panel $\hbox {pH}_i$ to $\hbox {pH}_e$ ratio for varying $\hbox {V}_c/\hbox {V}_C$ values (i.e. ratio of extracellular to cell volumes) when CA9 activity is turned on or off. In these simulations the extracellular environment is physically closed, i.e. the extracellular volume is unstirred and the diffusion of chemical species toward an “external reservoir” is not allowed.

As previously noted by Swietach et al.^11^$\hbox {pH}_i$ regulation is not affected by CA9 expression in isolated tumor cells, but its role becomes important when cells are grown as three-dimensional aggregates (tumor spheroids). When expressed by cells grown as tumor spheroids CA9 induces a near uniform intracellular pH throughout the structure^11^, an observation that was explained by diffusion-reaction modeling as follows: CA9 coordinates $\hbox {pH}_i$ spatially by facilitating $\hbox {CO}_2$ diffusion in the unstirred extracellular space of the spheroid^11^. This intriguing conclusion, supported by experimental evidence, suggests that CA9 activity becomes important for the control of $\hbox {pH}_i$ by tumor cells at critical sizes of the extracellular volume. We tested this hypothesis with our model, and the results are shown in Fig. 5.

The role of CA9 in $\hbox {pH}_i$ regulation starts to become important at the extracellular to cell volume ratio $\text {V}_c/\text {V}_C\approx 10^4$ and reaches a maximum at $\text {V}_c/\text {V}_C\approx 100$. It is important to note that we simulated cells that grow in a closed environment. This means that at small extracellular volumes the acidity of the environment becomes too high and $\hbox {pH}_i$ runs out of control (see also Fig. 4). However, the results in Fig. 5 show that when $\text {V}_c/\text {V}_C\approx 100$ and CA9 is active the extracellular pH at equilibrium is around 5.5 and $\hbox {pH}_i\approx 6.6$, well within the physiological range.

**Figure 6:**
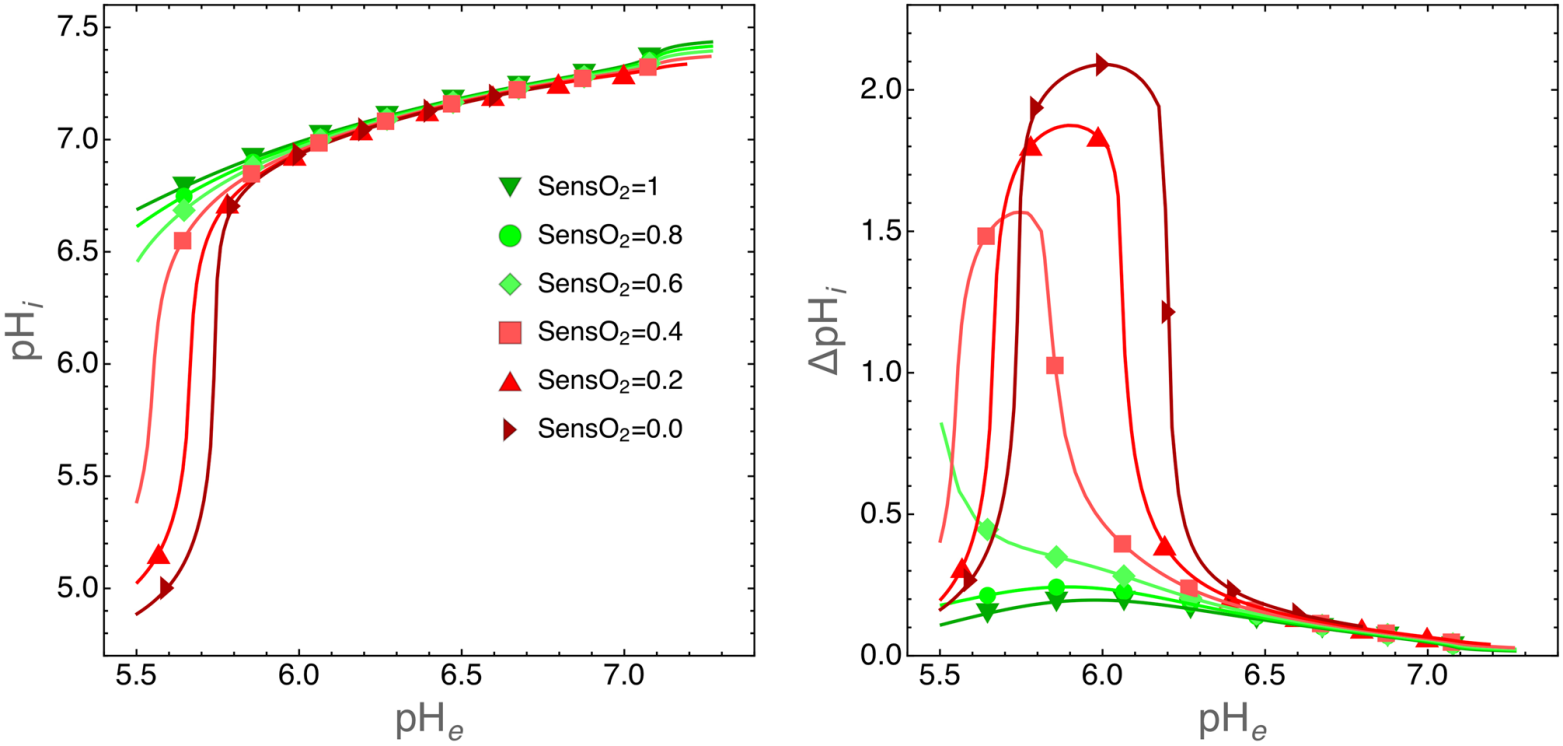
Role of CA9 on $\hbox {pH}_i$ regulation for cells grown in a small environment with decreasing oxygen levels. In these simulations the extracellular volume was set to $\hbox {V}_c=10^5\, \upmu \text {m}^3$ and cell radius to $\hbox {r}_C=6.5 \,\upmu \hbox {m}$ so that $\text {V}_c/\text {V}_C\approx 80$. Left panel: plot of $\hbox {pH}_i$ at equilibrium as the function of $\hbox {pH}_e$ for the indicated fractions of environmental $\hbox {O}_2$. Right panel: same simulations as those shown in the left panel, but here we plot $\Delta \text {pH}_i=\text {pH}_{i,\text {CA9=on}}-\text {pH}_{i\text {,CA9=off}}$, i.e. the difference in $\hbox {pH}_i$ when CA9 is turned on or off. This plot clearly shows the nonlinear character of CA9 activity in the regulation of $\hbox {pH}_i$.

Simulations in Fig. 5 do not take into account the oxygen levels in the tumor environment. As discussed above (see the “Methods” section) CA9 expression is regulated by hypoxia^22^ and thus it is interesting to investigate how $\hbox {pH}_i$ is regulated by cells growing in small environments, i.e. when the CA9 role is not negligible, and when $\hbox {O}_2$ levels are lower and lower. Figure 6 shows that when $\hbox {pH}_e\ge 5.8$, CA9 acts as a nonlinear $\hbox {pH}_i$ equalizer at any $\hbox {O}_2$ levels.

### The model as a tool for exploratory data analysis

Germ-line mutations that inactivate the von Hippel-Lindau (*vhl*) gene cause the VHL syndrome, a rare inherited disorder characterized mainly, but not only, by renal cancers^25,26^. The VHL protein drives ubiquitination and finally degradation of the hypoxia-inducible factor alpha (HIF) which in turn regulates a number of intracellular pathways that collectively confer resistance to hypoxia to cancer cells^25,26^. However, experimental findings suggest that both HIF-dependent and HIF-independent mechanisms are essential for VHL-mediated tumor suppressor effects^25,26^.

Stable transfection of 786-O renal cancer cells with a full-length human *vhl* gene significantly decreased proton and bicarbonate fluxes with respect to *vhl*-null cells in spite of increased or unaltered expression of ion transporters^27^. In particular, experiments showed that the rate of $\hbox {pH}_i$ change ($\hbox {dpH}_i/\hbox {dt}$) upon alkali or acid load was reduced to $\sim 25{-}45\%$ in $\hbox {VHL}^+$ cells with respect to $\hbox {VHL}^-$ cells. A number of control experiments were carried out to test possible effects of VHL proteins in these cells, but the effects of VHL protein on ion fluxes remained unexplained^27^. Here we modify our model to provide a possible interpretation of these experimental observations.

In the experiments with $\hbox {VHL}^+$ and $\hbox {VHL}^-$ cells, proton fluxes were measured in cells exposed to $\hbox {Cl}^-$-deprived solutions, during recovery from $\hbox {NH}_4^+$-induced cell acidification or subjected to hypertonic shock^27^. Simulations of $\hbox {NH}_4^+$-induced cell acidification and hypertonic shock would require major revision of the model to include a number of chemical, biochemical and morphological details such as, e.g., $\hbox {NH}_4\hbox {Cl}$ dissociation kinetics and intra- and extracellular flows of all involved ionic species, cell volume dynamics during osmotic shock and a detailed description of how cell shrinkage and swelling activate ions transport. In addition, quantitative information which is required to set the values of specific model parameters is not fully available, further hampering the development of specific detailed models. We therefore focus on cell treatments with $\hbox {Cl}^-$-deprived solutions.

**Figure 7:**
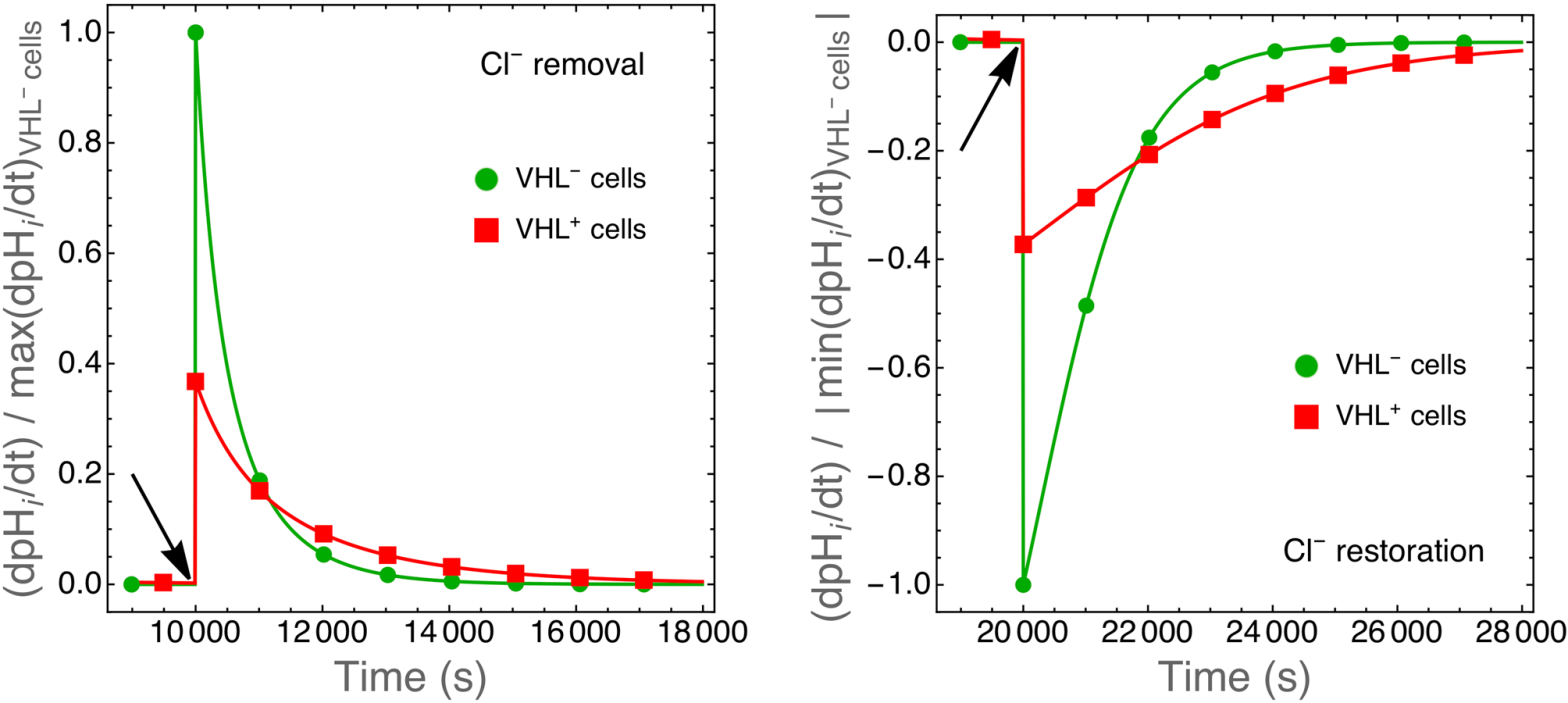
Rate of intracellular pH change in simulated $\hbox {VHL}^-$ and $\hbox {VHL}^+$ cells upon removal (left panel) and restoration (right panel) of environmental $\hbox {Cl}^-$ anions. Values have been normalized with respect to the maximum (left panel) or to the absolute value of the minimum (right panel) rate of $\hbox {pH}_i$ change calculated for $\hbox {VHL}^-$ cells. Simulations were run until $\hbox {pH}_i$ reached equilibrium. $\hbox {Cl}^-$ removal or restoration was modeled by suddenly (arrows) switching to 0 or to normal values, respectively, the rate of $\hbox {HCO3}^-$ efflux (Eq. 8, see the “Methods” section) as described in the text. The initial rate of $\hbox {pH}_i$ change in $\hbox {VHL}^+$ is reduced to $\sim 40\%$ of that of $\hbox {VHL}^-$ cells as observed in actual experiments.

The rationale behind cell treatment with $\hbox {Cl}^-$-deprived solutions was the discovery that VHL expression in 786-O renal cancer cells increased mRNA and protein levels of $\hbox {Cl}^-/\hbox {HCO}_3^-$ AE2 anion exchanger by 3.5 fold, although the apparent cell surface expression of AE2 was similar in $\hbox {VHL}^+$ and $\hbox {VHL}^-$ cells as evaluated by immunostaining^27^. The AE2 transporter exchange $\hbox {Cl}^-$ with $\hbox {HCO}_3^-$, and when the cells are exposed to $\hbox {Cl}^-$-deprived solutions $\hbox {Cl}^-$ can only exit from the cells thus forcing $\hbox {HCO}_3^-$ import^27^. In other words, the treatment makes an otherwise bidirectional transport unidirectional. Our simplified model takes into account only a generic unidirectional transporter that shuttle $\hbox {HCO}_3^-$ from the environment into the cell and that is described by Eq. 6 (see the “Methods” section). To model the AE2 exchanger we introduce one more equation to describe also the rate of $\hbox {HCO}_3^-$ efflux (see Eq. 8 in the “Methods” section). We then perturbed the system at equilibrium by suddenly switching to 0 the rate of $\hbox {HCO}_3^-$ efflux to model cells placed in $\hbox {Cl}^-$-deprived baths or switching it to normal values to model cells re-placed under standard environmental conditions (see Fig. 7).

The expression of many genes is altered in $\hbox {VHL}^+$ cells and VHL protein is known to affect several physiologic pathways^28^. Quantitative data are not fully available and therefore it is impossible with the present knowledge to reproduce the whole complex phenotype of these cells *in silico*. However, we note that among the physiologic pathways altered in 786-O cells expressing VHL proteins glycolysis and respiration are prominent^28^. Glycolysis was observed to be approximately one half of that measured for $\hbox {VHL}^-$ cells, a finding that was paralleled by a corresponding two fold downmodulation of glucose transporters, whereas respiration was found to be increased by a factor of two^28^. VHL expression was also observed to dramatically reduce (i.e. a $\sim 80-100$-fold change) lactate transport in other cell systems^29^. Our model can easily take into account the phenotype of $\hbox {VHL}^+$ cells as far as these pathways are concerned. We multiplied specific rates by appropriate factors: the rate of proton production ($\hbox {gH}^+$ in Eq. 11) was divided by 2 to model the reduced lactate/$\hbox {H}^+$ production by glycolysis; the rate of $\hbox {CO}_2$ production ($\hbox {gCO}_2$ in Eq. 11) was multiplied by 2 to model the increased respiration rate; finally the maximum rate of lactate transport through MCT transporters ($\nu _{\mathrm{maxMCT}}$ in Eq. 2) was divided by 80 to model the observed reduction of lactate transport.

**Figure 8:**
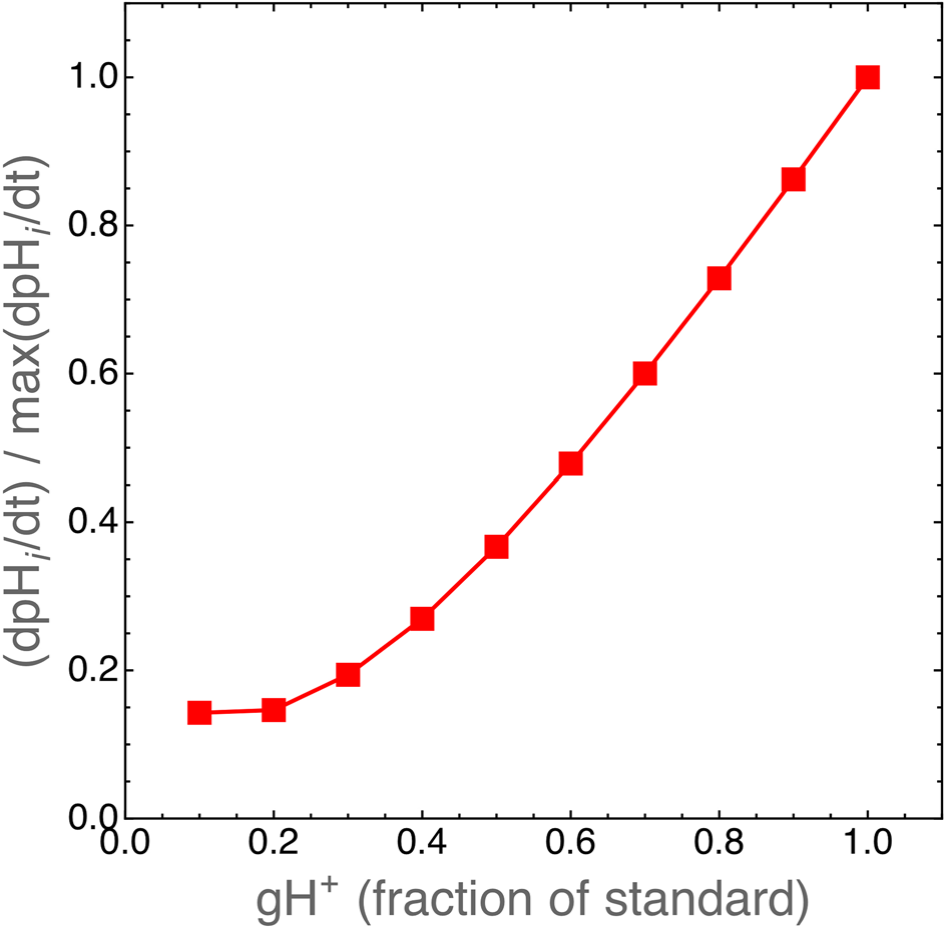
Reduced glycolytic rates in $\hbox {VHL}^+$ cells might explain the effect shown in Fig. 7. VHL protein expression was observed to downmodulate the expression of glucose transporters and to reduce the glycolytic rate, and hence lactate/$\hbox {H}^+$ production, in renal cancer cells^28^. Here we plot the rate of $\hbox {pH}_i$ change in simulated $\hbox {VHL}^+$ cells as the function of proton production rate through glycolysis (rate $\hbox {gH}^+$ in Eq. 11, see the “Methods” section and the Supplementary material). The standard value of $\hbox {gH}^+$ is calculated (see Eq. 11) as $\hbox {gAcL}\cdot (\hbox {MW}_\text {H}/\hbox {MW}_\text {AcL})$ where the value of gAcl is given in Table 1. When $\hbox {gH}^+=1$, $\hbox {dpH}_i/\hbox {dt}$ in $\hbox {VHL}^+$ cells is equal to that of $\hbox {VHL}^-$ cells.

The simulations show that the initial rate of intracellular pH change ($\hbox {dpH}_i/\hbox {dt}$) is reduced to $\sim 40 \%$ in $\hbox {VHL}^+$ cells with respect to $\hbox {VHL}^-$ cells (Fig. 7) in agreement with experimental observations^27^. As shown in Fig. 8, a reduced glycolytic rate is mainly responsible for this effect. This shows that the present model, although simplified, can still be adapted to simulate different cell phenotypes and used to suggest novel interpretation of otherwise paradoxical^27^ and yet unexplained experimental observations.

## Discussion

We have developed a biophysical model to explore the complex molecular mechanisms that allow tumor cells to regulate both intracellular and extracellular acidity, but we are not alone, other modeling efforts have tried to capture the essential features of the biochemical pathways that lead to acid homeostasis in tumor cells (see e.g.^30–33^). We have taken the remarkable models described in^32^ and^33^ as our starting point, because of their direct applicability to the analysis of experimental data. The former provides a fully tractable quantitative description of the interplay between $\hbox {H}^+$ and $\hbox {HCO}_3^-$ transporters with $\hbox {Na}^+/\hbox {K}^+$-ATPase and $\hbox {Na}^+$, $\hbox {K}^+$ and $\hbox {Cl}^-$ ion fluxes, while the latter investigates the interaction of MCT transporters and CA9. We go a few steps further and model the network of important paths that connect together cell metabolism and hypoxia with transport of $\hbox {H}^+$ and $\hbox {HCO}_3^-$ ions and CA9 activity (see Fig. 1). The coupling of ion transport mechanisms with metabolism and hypoxia is essential if we want to understand how tumor cells grow and shape their microenvironment, an interplay that is of fundamental importance for the adaptation and evolution of cancer cells within a solid tumor. As mentioned at the beginning of the Results section, we have developed a computer program that successfully reproduces the growth and the behavior of tumor cells in both liquid and solid cancers^6–8^. It is a lattice-free model that contains a rather detailed description of tumor cell metabolism and of the cell cycle, as well as many other biochemical and biophysical features (e.g. cell mechanics, cell division, etc.)^6–8^. This has already allowed us to characterize new biophysical properties of tumors and of their microenvironment^34–37^, but the program still contains an excindingly simplified description of how cells control their intracellular pH. The program has an incremental structure, and we add new parts as soon as they are independently validated. The present model is one of these parts, and once integrated in our previous software it will further increase its descriptive and predictive potential. We hope in this way to understand key biological features such as cell adaptation and evolution in tumor microenvironments and explore important aspects such as tumor cell resistance to therapies. Here we show that the present model can nonetheless be used as a tool for exploratory data analysis and for quantitative purposes.

We remark that with the model described here we are able to give a quantitative assessment of the importance of specific molecular mechanisms. For instance, simulations show that $\hbox {H}^+$ efflux from tumor cells dominates the control of intracellular acidity in normoxic environments, whereas $\hbox {HCO}_3^-$ import in hypoxic tumor areas (in our simulation where the fraction of oxygen decrease to 0.1 of standard values). Experiments have shown that in *in vivo* tumor micro-environments oxygen reaches 10% of its normal value at a distance of $\approx 150 \,\upmu \hbox {m}$, i.e. $\approx 10$ cell diameters, from blood vessels^38^. Thus, within this short distance the control of $\hbox {pH}_i$ is attained by tumor cells through a switch from $\hbox {H}^+$ export to $\hbox {HCO}_3^-$ import pathways. This observation gives further support to recent work that has shown that inhibition of $\hbox {HCO}_3^-$ fluxes inhibits the growth of experimental tumors by increasing intracellular acidity and cell death^39^. When we recall that the hypoxic regions are those where tumor cells show higher resistance to therapies, such as e.g. radiotherapy, then we see that approaches that aim at inhibiting $\hbox {HCO}_3^-$ fluxes would target the very cells that colonize the inner tumor regions and that would otherwise be resistant to therapies, and improve cancer control.

Finally, the model singles out the important role of CA9. The simulations show that CA9 acts as a nonlinear $\hbox {pH}_i$ equalizer at any $\hbox {O}_2$ level in cells that grow in acidic extracellular environments. This result is in agreement with the experimental observations by Swietach and colleagues^11^, collected with tumor spheroids. They observed near-uniform $\hbox {pH}_i$ values throughout the spheroid structure due to CA9 activity in spheroids grown up to $\approx 500 \,\upmu \hbox {m}$ diameter. It has long been recognized that tumor spheroids of this size show steep gradients of oxygen with fractions that go as far down as 0 at the center of the spheroid^40^. Our simulations show that this is due to the concerted action of CA9 and of hypoxia that up-regulates CA9 expression. These two mechanisms collectively help cells to keep their intracellular pH under control because of increased $\hbox {HCO}_3^-$ production followed by $\hbox {HCO}_3^-$ import through THCO3 transporters.

## Conclusion

While tumor cell adaptation and survival to extreme microenvironments are key concepts in oncogenesis^1–3^, we remark that acid homeostasis is central to cellular adaptation in a much wider context. Active transport of acid/base equivalents across cell membranes into the extracellular spaces may cause transient and rapid changes of microenvironmental and cellular pHs like those observed for other ions involved in cell signalling. Indeed, pH transients have been shown to be important in intra- and inter-cellular communication in the nervous system and are known to affect a number of essential functions, like e.g. neuronal excitability and synaptic transmission^41^. This in turn implies that animal cells could sense and adapt to pH changes. The underlying molecular mechanisms are still not well understood, but the role of G-protein coupled receptors in proton sensing is increasingly investigated also in relation to pathological conditions, besides cancer, that result in an increased extracellular acidity, such as infarction and inflammation^42^. We conclude that our model can be used as an essential building block of more comprehensive *in silico* research on solid tumors^43^, but it may also help understanding how other cells can sense and dynamically adapt to pH changes.

## Methods

### Bicarbonate buffer and initial conditions

Central to the whole scheme of reactions shown in Fig. 1 is the hydration of $\hbox {CO}_2$. It is well known that at physiologic temperature (i.e. $\sim 37\,^\circ \hbox {C}$) carbonic acid dissociates very quickly and represents less than 0.5% of the total carbon dioxide and bicarbonate ion^44^. Thus, the hydration of $\hbox {CO}_2$ can be approximated by the following chemical reaction:

$$\begin{aligned} \text {CO}_2 + \text {H}_2\hbox {O}{{\mathop {\rightleftharpoons}\limits ^{k_2}_{k_1}}} \text {H}^+ + \text {HCO}_3^- \end{aligned}$$

The values of the two rate constants $k_1$ and $k_2$ have been determined in cells under standard culture conditions in two independent experiments with good agreement^11,18^. We take the values in^18^: $k_1\simeq 0.144\,\hbox {s}^{-1}$ and $k_2\simeq 1.9 \cdot 10^5\,\hbox {M}^{-1}\,\hbox {s}^{-1}$.

We compare model outputs with experimental data obtained with cell cultures *in vitro*, in a standard atmosphere at $37\,^\circ \hbox {C}$ and 5% $\hbox {CO}_2$ at 1 atm pressure. To compute the initial density of $\hbox {CO}_2$ dissolved in water under these conditions we use Henry’s law $c=k(T)P$ where *c* is the molar concentration of the gas in water, *P* the pressure and *k*(*T*) is a function of temperature

$$\begin{aligned} k(T)=k^\Theta \exp {\left[ -\frac{\Delta _\text {sol}\,k}{R}\left( \frac{1}{T}-\frac{1}{T^\Theta }\right) \right] } \end{aligned}$$

with $T^\Theta =298.15\,\text {K}$, $k^\Theta =3.3\cdot 10^{-4} {\text {mol}}\;{\text {m}^{-3}\;\text {Pa}^{-1}}$ and $-\frac{\Delta _\text {sol}\,k}{R}=2400\,\text {K}$ (see ref.^45^ for further details); we find that the initial density of $\hbox {CO}_2$ in cell medium under standard culture conditions is:

$$\begin{aligned} \rho _{\mathrm{CO}_2}=5.39\cdot 10^{-5}\,\frac{\text {pg}}{\upmu \text {m}^3} \end{aligned}$$

Finally, given the $\hbox {CO}_2$ concentration we find the density of $\hbox {HCO}_3^-$ ions from the Henderson-Hasselbach equation:

$$\begin{aligned} \text {pH}=\text {pKa}+\log _{10}{\left( \frac{[\text {HCO}^-_3]}{[\hbox {CO}_2]}\right) } \end{aligned}$$

where $$\text {pKa}=-\log _{10}{({\text {k}_1}/{\text {k}_2})}\simeq 6.12$$.

Where not otherwise specified, we fixed the standard intracellular and extracellular pH at 7.4, which determines the initial value of the molar concentration of $\hbox {H}^+$ ions inside and outside the cells.

### $\hbox {CO}_2$ diffusion through the cell membrane

Given the assumptions above, the component of $\hbox {CO}_2$ normal to the cell membrane is described by the Fick’s first law:

$$\begin{aligned} J_{1\rightarrow 2}=-P_{\mathrm{M,CO}_2}\cdot (C_2-C_1) \end{aligned}$$

where $J_{1\rightarrow 2}$ is the flux from 1 to 2 in units of concentration over time and surface area $S_C$, $P_{\mathrm{M,CO}_2}$ is the permeability of the carbon dioxide and $C_i$ is the concentration of $\hbox {CO}_2$ in the *i*-th volume. Since we model cells grown in an incubator at constant $\hbox {CO}_2$ pressure, the $\hbox {CO}_2$ concentration can reach values far from equilibrium only inside cells because of the oxygen consumption by cell metabolism and of the equivalent $\hbox {CO}_2$ production. This means that in the present model there is only a net outward flux of carbon dioxide from cells to the environment. Thus, the net flux of $\hbox {CO}_2$ due to diffusion is:

1 $$\begin{aligned} \left. \frac{dm_{\mathrm{CO}_2\text {,C}}}{dt}\right| _\text {diff}=-J_{1\rightarrow 2}\cdot \text {MW}_{\mathrm{CO}_2} \cdot S_C=P_{\mathrm{M,CO}_2}\left( \frac{m_{\mathrm{CO}_2,c}}{V_c}-\frac{m_{\mathrm{CO}_2,C}}{V_C}\right) S_C \end{aligned}$$

### MCT transporters

The MCTs are a family of bidirectional $\hbox {H}^+$ and lactate co-transporters expressed at the cell membrane and their activity has been shown to depend on the pH values on both sides of the cell membrane (see refs.^6–8^ and references therein). We model their activity with parameter values extrapolated from experimental observations^6–8^ and we use the following equations and parameters to describe the rate of transport of $\hbox {H}^+$ inside and outside the cell:

2 $$\begin{aligned} \nu _{\mathrm{MCT}}^{\text {out}\rightarrow \text {in}}&=\text {a2c}_{\mathrm{H}}\cdot \frac{\nu _{\mathrm{maxMCT}}\cdot m_{\mathrm{H}^+,c}}{V_c K_{\mathrm{mMCT}} + m_{\mathrm{H}^+,c}}\nonumber \\ \nu _{\mathrm{MCT}}^{\text {in}\rightarrow \text {out}}&=\text {c2a}_{\mathrm{H}}\cdot \frac{\nu _{\mathrm{maxMCT}}\cdot m_{\mathrm{H}^+,C}}{V_C K_{\mathrm{mMCT}} + m_{\mathrm{H}^+,C}} \end{aligned}$$

where $\nu _{\mathrm{maxMCT}}=V_{\mathrm{maxAcL}}\cdot \frac{\text {MW}_{\mathrm{H}}}{\text {MW}_{\mathrm{AcL}}}\cdot S_C$, $K_{\mathrm{mMCT}}=K_{\mathrm{mAcL}}\cdot \frac{\text {MW}_{\mathrm{H}}}{\text {MW}_{\mathrm{AcL}}}$ and where the ratio of molecular weights is used to rescale the equations from concentrations to masses.

In Eq. 2, $\hbox {a2c}_{\mathrm{H}}$ and $\hbox {c2a}_{\mathrm{H}}$ depend, respectively, on extracellular and intracellular pH, and phenomenologically describe the dependency of MCT transport activity on acidity (for a complete analysis see^6–8^):

3 $$\begin{aligned} \text {a2c}_{\mathrm{H}}&=2-\tanh (\text {a2c}_{\mathrm{H}\_\mathrm{slope}}\cdot \text {pH}_c - \text {a2c}_{\mathrm{H}\_\mathrm{thr}})\nonumber \\ \text {c2a}_{\mathrm{H}}&=2-\tanh (\text {c2a}_{\mathrm{H}\_\mathrm{slope}}\cdot \text {pH}_C - \text {c2a}_{\mathrm{H}\_\mathrm{thr}}) \end{aligned}$$

### NHE transporters

Sodium-hydrogen exchangers (NHE) are membrane transport proteins that exploit the influx of $\hbox {Na}^+$ to export $\hbox {H}^+$ ions. The sodium concentration gradient is maintained by the ATP-dependent $\hbox {Na}^+/\hbox {K}^+$ pump^19,46^ so that the activity of NHE indirectly depends on ATP availability. This implies that as long as ATP is available the flux of $\hbox {H}^+$ due to NHE is essentially unidirectional. It has also been reported that NHE activity is inhibited by hypoxia^10,19^ and that, in the long-term, hypoxia inhibits the expression of NHE proteins. Energy and oxygen tune NHE activity and as in the previous model of tumor cell metabolism and growth^6–8^, here we take into account these regulatory circuits by means of the two variables SensATP and $\hbox {SensO}_2$ that assume real values in the interval [0, 1].

Experimental observations indicate that NHE activity is described by a Hill equation^9,47,48^ and hence the unidirectional flux of $\hbox {H}^+$ from the cell to the environment due to NHE transport is modeled by the equation:

4 $$\begin{aligned} \nu _{\mathrm{NHE}}^{\text {in}\rightarrow \text {out}}=\text {SensATP}\cdot \text {SensO}_2 \cdot \text {fpHe}_{\mathrm{NHE}}\cdot \frac{\nu _{\mathrm{maxNHE}}\cdot m^h_{\mathrm{H}^+,C}}{(V_C \cdot \text {MW}_{\mathrm{H}}\cdot K_{\mathrm{mNHE}})^h+m^h_{\mathrm{H}^+,C}} \end{aligned}$$

where $\nu _\text {maxNHE}=\text {V}_\text {maxNHE}\cdot S_C$ and $\hbox {fPHe}_{\mathrm{NHE}}$ is a phenomenological function that tunes the activity of NHE transport as a function of extracellular pH:

5 $$\begin{aligned} \text {fpHe}=\frac{1}{2}\left( 1+\frac{\text {pH}_{\mathrm{e}}-\text {pH}_0}{\lambda +\left|\text {pH}_{\mathrm{e}}-\text {pH}_0 \right|}\right) \end{aligned}$$

Indeed, it has been observed that extracellular acidity enhances $\hbox {H}^+$ transport through NHE^9,19,49^. In the Supplementary Material we discuss how we fix parameter values and define the function fPHe on the basis of experimental observations.

### Transport of bicarbonate ions

As discussed above, we model the activity of a generic bicarbonate ion importer (THCO3). The $\hbox {Na}^+$-dependent $\hbox {Cl}^-/\hbox {HCO}_3^-$ exchanger appears to dominate $\hbox {HCO}_3^-$ fluxes in tumor cells^9,10^, and therefore we take this transporter as a reference to set the values of parameters and fix general biochemical characteristics. This is an important part of the model, because it has been shown that tumor cells do actively import $\hbox {HCO}_3^-$ ions to buffer their internal pH^9,10^, and that this is a common property of different cancer cells. Experimental studies have demonstrated that $\hbox {HCO}_3^-$ import is regulated by both intracellular and extracellular pH but not by hypoxia and that the transport follows a simple Michaelis-Menten kinetics. In the scientific literature there are no indications, as far as we can tell, that $\hbox {HCO}_3^-$ transport depends on ATP availability. However, just as observed for proton export by NHE transporters, $\hbox {HCO}_3^-$ transport proceeds by parallel fluxes of ions, like $\hbox {Na}^+$ and $\hbox {Cl}^-$, along their electrochemical gradients that are actively maintained by cells through energy-consuming paths. Thus, it is likely that even $\hbox {HCO}_3^-$ transport is controlled by ATP availability, albeit indirectly. On the basis of these considerations we model $\hbox {HCO}_3^-$ import as follows:

6 $$\begin{aligned} \nu _\text {THCO3}^{\text {out}\rightarrow \text {in}}=\text {SensATP}\cdot \text {fpHe}_{\mathrm{THCO3}}\cdot \text {fpHi}_{\mathrm{THCO3}}\cdot \frac{\nu _{\mathrm{maxTHCO3}}\cdot m_{\mathrm{HCO}_{3}^-\text {,c}}}{V_{c}\cdot \text {MW}_{\mathrm{HCO3}}\cdot K_{\mathrm{mHCO3}}+ m_{\mathrm{HCO}_{3}^-\text {,c}}} \end{aligned}$$

where $\nu _\text {maxTHCO3}=\text {V}_\text {maxTHCO3}\cdot S_C$ and the two functions $\hbox {fpHe}_\text {THCO3}$ and $\hbox {fpHi}_\text {THCO3}$ phenomenologically describe how $\hbox {HCO}_3^-$ import is affected by extracellular and intracellular pH, respectively. These functions have been fit to actual experimental data (see the Supplementary Material) and are modeled by the following equations:

7 $$\begin{aligned} \text {fpHi}_\text {THCO3}&=\frac{1}{2}\{1+\tanh {[\gamma _\text {THCO3}\cdot (\text {pHi}_\text {0,THCO3}-\text {pH}_\text {i})]}\}\nonumber \\ \text {fpHe}_\text {THCO3}&=\frac{1}{2}\{1+\tanh {[\lambda _\text {THCO3}\cdot (\text {pH}_\text {e}-\text {pHe}_\text {0,THCO3})]}\} \end{aligned}$$

In *in silico* experiments with $\hbox {VHL}^+$ and $\hbox {VHL}^-$ cells we make $\hbox {HCO}_3^-$ transport bidirectional by considering $\hbox {HCO}_3^-$ efflux from cells as follows:

8 $$\begin{aligned} \nu _\text {THCO3}^{\text {in}\rightarrow \text {out}}=\text {SensATP}\cdot \text {fpHe}_{\mathrm{THCO3}}\cdot \text {fpHi}_{\mathrm{THCO3}}\cdot \frac{\nu _{\mathrm{maxTHCO3}}\cdot m_{\mathrm{HCO}_{3}^-\text {,C}}}{V_{C}\cdot \text {MW}_{\mathrm{HCO3}}\cdot K_{\mathrm{mHCO3}}+ m_{\mathrm{HCO}_{3}^-\text {,C}}} \end{aligned}$$

### Activity of Carbonic Anhydrase 9

The enzyme CA9 is expressed by cells of many different solid tumors, and in general its expression correlates with cancer aggressiveness and poor therapeutic outcome^11–13^. It is a membrane-tethered enzyme and it is mainly found at the external surface of cells where it catalyses the hydration of $\hbox {CO}_2$^11–13^. Importantly, its expression is regulated by hypoxia and indeed CA9 is a marker of hypoxia^22^. Again, experimental observations show that CA9 activity follows a Michaelis-Menten kinetics. Thus:

9 $$\begin{aligned} \nu _\text {CA9}=\text {h}_\text {CA9}\cdot \frac{\nu _\text {maxCA9}\cdot m_{\mathrm{CO}_2\text {,c}}}{V_{c}\cdot \text {MW}_\text {CO2}\cdot K_\text {mCA9}+m_{\mathrm{CO}_2\text {,c}}} \end{aligned}$$

where where $\nu _\text {maxCA9}=\text {V}_\text {maxCA9}\cdot S_C$ and $\hbox {h}_\text {CA9}$ is a phenomenological functions that describe how hypoxia tunes CA9 expression:

10 $$\begin{aligned} \text {h}_\text {CA9}=3+2\cdot \tanh {\left( -\delta _\text {CA9}\cdot \text {SensO2}\right) } \end{aligned}$$

This is a function of the fraction of available oxygen which, in our model, is defined by SensO2, and it describes the fold change in CA9 expression as observed in actual experiments (see the Supplementary Material).

### The full model and its numerical integration

The full model is represented by the following set of differential equations:

11 $$\begin{aligned} \frac{dm_{\mathrm{CO}_2\text {,C}}}{dt}&=\text {gCO}_2-k_1\cdot m_{\mathrm{CO}_2\text {,C}}+k_2 \cdot m_{\mathrm{H}^+\text {,C}}\cdot m_{\mathrm{HCO}_3^-\text {,C}}\cdot \frac{10^3\cdot \text {MW}_{\mathrm{CO}_2}}{V_C\cdot \text {MW}_{\mathrm{H}}\cdot \text {MW}_\text {HCO3}}-J_{C\rightarrow c}\cdot S_C\cdot \text {MW}_{\mathrm{CO}_2}\nonumber \\ \frac{dm_{\mathrm{H}^+\text {,C}}}{dt}&=\text {gH}^+ +k_1\cdot m_{\mathrm{CO}_2\text {,C}}\cdot \frac{\text {MW}_\text {H}}{\text {MW}_{\mathrm{CO}_2}}-k_2 \cdot m_{\mathrm{H}^+\text {,C}}\cdot m_{\mathrm{HCO}_3^-\text {,C}}\cdot \frac{10^3}{V_C\cdot \text {MW}_\text {HCO3}}\nonumber \\&\quad -\,\nu _{\mathrm{MCT}}^{\text {in } \rightarrow \text {out}}+\nu _{\mathrm{MCT}}^{\text {out } \rightarrow \text { in}}-\nu _{\mathrm{NHE}}^{\text {in } \rightarrow \text { out}}\nonumber \\ \frac{m_{\mathrm{HCO}_3^-\text {,C}}}{dt}&=k_1\cdot m_{\mathrm{CO}_2\text {,C}}\cdot \frac{\text {MW}_\text {HCO3}}{\text {MW}_{\mathrm{CO}_2}}-k_2 \cdot m_{\mathrm{H}^+\text {,C}}\cdot m_{\mathrm{HCO}_3^-\text {,C}}\cdot \frac{10^3}{V_C\cdot \text {MW}_\text {H}}+\nu _{\mathrm{THCO3}}^{\text {out } \rightarrow \text { in}}\nonumber \\ \frac{dm_{\mathrm{CO}_2\text {,c}}}{dt}&=0\nonumber \\ \frac{dm_{\mathrm{H}^+\text {,c}}}{dt}&=k_1\cdot m_{\mathrm{CO}_2\text {,c}}\cdot \frac{\text {MW}_\text {H}}{\text {MW}_{\mathrm{CO}_2}}-k_2 \cdot m_{\mathrm{H}^+\text {,c}}\cdot m_{\mathrm{HCO}_3^-\text {,c}}\cdot \frac{10^3}{V_c\cdot \text {MW}_\text {HCO3}}+\nu _{\mathrm{MCT}}^{\text {in } \rightarrow \text { out}}-\nu _{\mathrm{MCT}}^{\text {out } \rightarrow \text { in}}\nonumber \\&\quad +\,\nu _{\mathrm{NHE}}^{\text {in } \rightarrow \text { out}}+\nu _{\mathrm{CA9}}\cdot \frac{\text {MW}_\text {H}}{\text {MW}_{\mathrm{CO}_2}}\nonumber \\ \frac{m_{\mathrm{HCO}_3^-\text {,c}}}{dt}&=k_1\cdot m_{\mathrm{CO}_2\text {,c}}\cdot \frac{\text {MW}_\text {HCO3}}{\text {MW}_{\mathrm{CO}_2}}-k_2 \cdot m_{\mathrm{H}^+\text {,c}}\cdot m_{\mathrm{HCO}_3^-\text {,c}}\cdot \frac{10^3}{V_c\cdot \text {MW}_\text {H}}-\nu _{\mathrm{THCO3}}^{\text {out } \rightarrow \text { in}}\nonumber \\&\quad +\,\nu _{\mathrm{CA9}}\cdot \frac{\text {MW}_\text {HCO3}}{\text {MW}_{\mathrm{CO}_2}} \end{aligned}$$

where $\hbox {gH}^+ =\hbox {gAcL}\cdot \hbox {MW}_\text {H}/\hbox {MW}_\text {AcL}$ and $\hbox {gCO}_2=\hbox {qO}_2 \cdot \hbox {MW}_{\mathrm{CO}_2}/\hbox {MW}_{\mathrm{O}_2}$ are, respectively, the rates of $\hbox {H}^+$ and $\hbox {CO}_2$ production that are proportional to the rate of lactate production gAcL and oxygen consumption $\hbox {qO}_2$ as defined in our previous work^6–8^, and all the other rates, and regulatory functions, are given in equations 1–10. The multiplicative factor $10^3$ that appears in the right-hand side of equations 11 above comes from the conversion of standard molar concentration units to the units used here where masses are expressed in pg and volumes in $\upmu \text {m}^3$.

In *in silico* experiments with $\hbox {VHL}^+$ and $\hbox {VHL}^-$ cells, where $\hbox {HCO}_3^-$ transport is bidirectional, the differential equations in the set 11 that describe $\hbox {HCO}_3^-$ kinetics were modified as follows:

$$\begin{aligned} \frac{m_{\mathrm{HCO}_3^-\text {,C}}}{dt}&=k_1\cdot m_{\mathrm{CO}_2\text {,C}}\cdot \frac{\text {MW}_\text {HCO3}}{\text {MW}_{\mathrm{CO}_2}}-k_2 \cdot m_{\mathrm{H}^+\text {,C}}\cdot m_{\mathrm{HCO}_3^-\text {,C}}\cdot \frac{10^3}{V_C\cdot \text {MW}_\text {H}}+\nu _{\mathrm{THCO3}}^{\text {out } \rightarrow \text { in}}-\nu _{\mathrm{THCO3}}^{\text {in } \rightarrow \text { out}}\\ \frac{m_{\mathrm{HCO}_3^-\text {,c}}}{dt}&=k_1\cdot m_{\mathrm{CO}_2\text {,c}}\cdot \frac{\text {MW}_\text {HCO3}}{\text {MW}_{\mathrm{CO}_2}}-k_2 \cdot m_{\mathrm{H}^+\text {,c}}\cdot m_{\mathrm{HCO}_3^-\text {,c}}\cdot \frac{10^3}{V_c\cdot \text {MW}_\text {H}}-\nu _{\mathrm{THCO3}}^{\text {out } \rightarrow \text { in}}+\nu _{\mathrm{THCO3}}^{\text {in } \rightarrow \text { out}}\\&\quad +\,\nu _{\mathrm{CA9}}\cdot \frac{\text {MW}_\text {HCO3}}{\text {MW}_{\mathrm{CO}_2}} \end{aligned}$$

The system of differential equations 11 is nonlinear and stiff because it incorporates processes with different kinetics, from the fast kinetics of $\hbox {CO}_2$ hydration and diffusion to the relatively slow kinetics of ion transport and enzyme activity. The system cannot be solved analytically and appropriate numerical approaches are required. We previously investigated this aspect within the context of complex large-scale biophysical models^50^ and found that the implicit Euler method is well-suited for the numerical integration of models of this kind. We solved the discretized system of differential equation 11 using the implicit Euler algorithm followed by the Newton-Raphson method to solve numerically the resulting system of nonlinear equations. The code has been implemented in C++ using the computational framework provided by the GNU Scientific Library^51^. We used the standard Newton-Raphson solver *gsl_multiroot_fsolver_dnewton* and the *gsl_multiroot_test_residual* library to test the convergence of the algorithm (threshold $\epsilon <10^{-6}$) within a maximum number of iterations fixed at $N_{\mathrm{max}}=1000$.

## Supplementary information

Supplementary information.
