# Supplementary material for: The control of acidity in tumor cells: a biophysical model
Source: Sci Rep. 2020 Aug 12;10:13613. doi: 10.1038/s41598-020-70396-1 (PMC7423962; doi:10.1038/s41598-020-70396-1)
Supplement: Supplementary file 1 — Supplementary information. [file 41598_2020_70396_MOESM1_ESM.pdf]

Supplementary Material

The control of acidity in tumor cells: a biophysical model

N. Piasentin [4][1], E. Milotti[2], and R. Chignola[3,*]

[1,2]Department of Physics, University of Trieste, Via Valerio 2, I-34127 Trieste, Italy
[3]Department of Biotechnology, University of Verona, Strada Le Grazie 15 - CV1, I-37134 Verona, Italy

In this document we detail the estimates of model parameters whenever not specified in the main text. In the Appendix we list the C++ code used for the simulations described in the Results section in the main text.

Rates of H^+ and CO_2 production

The rates of H^+ and CO_2 production are obtained from the metabolic model described in refs. (1, 2). To compute the rate of H^+ production by tumor cells we take into account the previously determined average rate of lactic acid production gAcL$\simeq 3.8 \cdot 10^{-19}$ kg s^{-1} (1). Under physiological conditions this acid completely dissociates to H^+ and lactate ions (2). Since these chemical species are in 1:1 molar ratio, the rate of H^+ production gH simply writes:

$$\mathrm{gH} = \mathrm{gAcL}\frac{\mathrm{MW_H}}{\mathrm{MW_{AcL}}}$$

where $\mathrm{MW_H} = 1$ g mol^{-1} and $\mathrm{MW_{AcL}} = 90$ g mol^{-1} are the molecular weights of H^+ and lactic acid.

Complete glucose oxidation requires 6 moles of O_2 per mole of glucose and in this reaction 6 moles of CO_2 are produced. Thus, if the respective rates of O_2 consumption and CO_2 production are qO_2 and gCO_2, we find:

$$\mathrm{gCO_2} = \mathrm{qO_2}\frac{\mathrm{MW_{CO_2}}}{\mathrm{MW_{O_2}}}$$

where $\mathrm{MW_{O_2}} = 32$ g mol^{-1} and $\mathrm{MW_{CO_2}} = 44$ g mol^{-1} are the molecular weights of O_2 and CO_2. Our previous work showed that for tumor cells on average $\mathrm{qO_2} \simeq 3.5 \cdot 10^{-20}$ kg s^{-1}.

Determination of parameters' values for NHE transporters

At least three independent experimental works confirmed that the activity of NHE transporters is described by a Hill equation (see also the main text) with exponent > 2 (3–5). In addition it has been reported that H^+ transport by NHE is inhibited by extracellular acidity (3).

To determine the values of parameters in equations that describe NHE transporters and their regulation by extracellular acidity we used the data in Fig.1, panel Ei, in ref. (3). The data have been obtained with careful measurements of H^+ fluxes in HCT116 cells (a human colorectal cancer cell line) with varying intracellular (pH_i) and extracellular (pH_e) pH. We redraw these data in figure S1.

We fitted these data with the following Hill equation:

$$\frac{dm_{\mathrm{H^+},C}}{dt} = \mathrm{V_{max}} \cdot \frac{[\mathrm{H^+}]_C^h}{K_\mathrm{m}^h + [\mathrm{H^+}]_C^h} \tag{1}$$

where square brackets denote molar concentrations, the subscript C is used for intracellular chemical species, $\mathrm{V_{max}}$ and K_m are the Michealis-Menten parameters and h is the Hill coefficient. Nonlinear fits were weighted with experimental errors and we used the χ^2/df statistics (df=degrees of freedom) to determine the goodness of the fits.

Best fit parameters values are listed in table S1. We take the average value of both K_m and h parameters calculated from the values shown in table S1, i.e. $K_\mathrm{m} = 0.1958 \pm 0.0124$ μM and $h = 2.67 \pm 0.15$, and the value of $\mathrm{V_{max}}$ estimated at $\mathrm{pH}_e = 7.4$. We compute the maximum ion flux per surface unit as $\mathrm{V_{max}}/\mathrm{S}_C$ where S_C is the cell surface. The reported radius of HCT116 cells is $\mathrm{r}_C = 6.55 \pm 0.14$ μm (3). We approximate the cell to a sphere and finally obtain the $\mathrm{V_{maxNHE}}$ value reported in Table 1 in the main text.

[4]current address: Department of Chemical and Process Engineering, University of Surrey, GU2 7XH, and Unilever R&D Colworth, Colworth Park, Sharnbook, Bedford MK44 1LQ, UK.

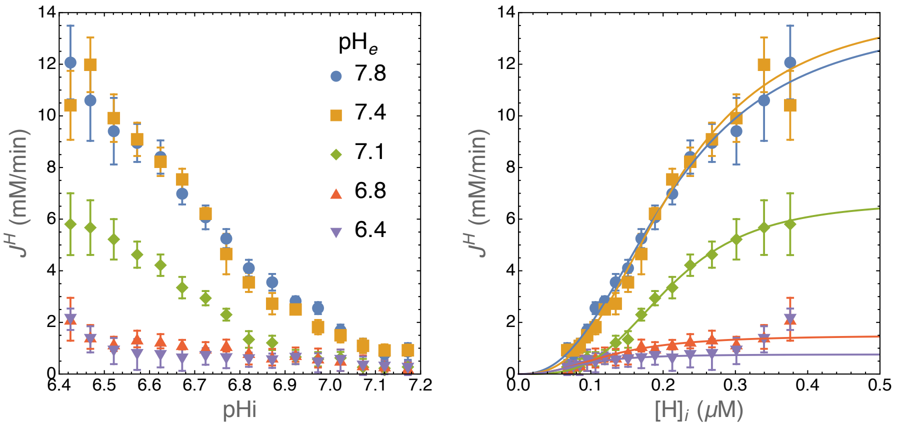

Figure S1: Fit of experimental data with equation 1. Left panel: the data taken in measurements of H^+ fluxes in HCT116 cells with varying pH$_i$ and pH$_e$, redrawn from ref. (3). Right panel: same data as in the left panel, but in this case the x-axis has been converted from pH$_i$ to intracellular H^+ concentration units. The lines show the best fits with the nonlinear Hill equation 1.

Table S1: Parameter values from nonlinear fits of the data in figure S1 with equation 1

pH$_e$	V$_{max}$(mM/min)	K_m (μM)	h	χ^2/df
7.8	13.99±2.18	0.2096±0.0277	2.47±0.25	1.22
7.4	14.31±1.70	0.2123±0.0199	2.71±0.22	1.24
7.1	6.73±1.18	0.2086±0.0242	3.44±0.53	1.20
6.8	1.51±0.46	0.1297±0.0438	2.43±1.30	1.24
6.4	0.76±0.42	0.0898±0.0473	2.68±3.81	1.32

To show how the V$_{max}$ of NHE transporters varies with pH$_e$ we plot the values listed in table S1 in figure S2. V$_{max}$ values were divided by the maximum observed value (i.e. V$_{max}$ estimated at pH$_e$=7.4) and then fitted with the following equation:

$$\mathrm{fpHe} = \frac{1}{2}\left(1 + \frac{\mathrm{pH_e} - \mathrm{pH_0}}{\lambda + |\mathrm{pH_e} - \mathrm{pH_0}|}\right) \tag{2}$$

Equation 2 describe how extracellular pH affects the activity of NHE transporters by reducing the maximal rate of H^+ transport by the fraction fpH$_e$. Fit of the values in figure S2 with equation 2 yielded the following values for estimated parameters (χ^2/df = 1.85): $\mathrm{pH}_{0,\mathrm{NHE}} = 7.10 \pm 0.01$, $\lambda_{\mathrm{NHE}} = 0.0759 \pm 0.0258$.

Determination of parameters' values for THCO3 transporters

We used the data in Fig.2 in (6) which show the dependence of proton fluxes in MGH U1 cells (a human bladder carcinoma cell line) on the extracellular concentration of bicarbonate ions. In these experiments the flux of protons is defined as the time variation of the product of pH$_i$ and the buffering capacity of the cells (6). The data follow the Michaelis-Menten kinetics and thus:

$$\frac{dm_{\mathrm{H^+},C}}{dt} = \mathrm{V_{max,H}} \cdot \frac{[\mathrm{HCO_3^-}]_c}{K_\mathrm{m} + [\mathrm{HCO_3^-}]_c} \tag{3}$$

where, as usual, the square brackets denote molar concentrations and where the subscript C denotes the intracellular environment and c the extracellular one. Fitting the experimental data with equation 3 we find the following values for the Michaelis-Menten parameters (χ^2/df = 1.47, see figure S3): $\mathrm{V_{max,H}} = 9.12 \pm 0.41$ mM/min, $K_\mathrm{m} = 7.38 \pm 0.77$ mM. A fit of the same data with a Hill equation returned a value of 0.94 ± 0.09, i.e. $\simeq 1$, for the exponent, further indicating that the activity of bicarbonate transporters is not governed by Hill kinetics.

Equation 3 is rather unusual because it relates two different quantities, namely the molar concentrations of protons and of bicarbonate ions. Recalling that for a generic chemical species the molar concentration is related to mass by $[\mathrm{X}] = m_\mathrm{X}/(V \cdot \mathrm{MW_X})$

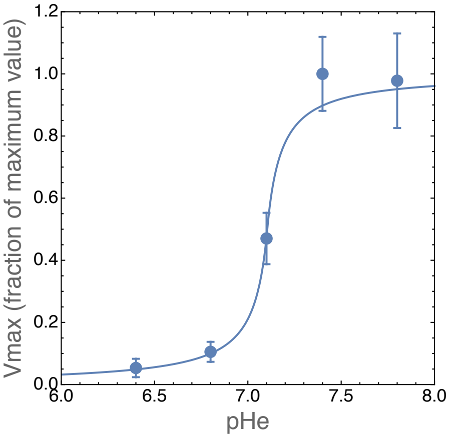

Figure S2: Plot of V_{max} values in table S1 (normalized with respect to the maximum reported value) as the function of extracellular pH. The line is the best fit with equation 2.

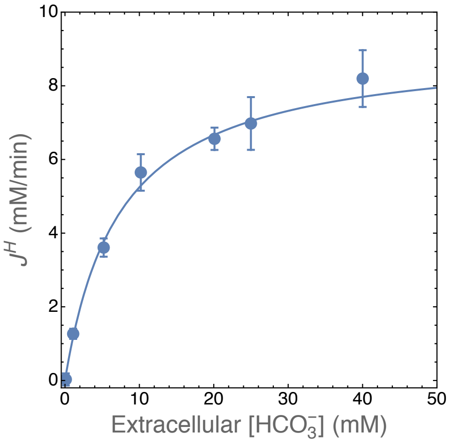

Figure S3: H^+ cell fluxes in MGH U1 cells as the function of extracellular concentration of HCO_3^- ions. Data have been redrawn from ref. (6). The line shows the best fit of experimental data with equation 3.

where V is the volume of the solution, then the left-hand side of equation 3 can be written as:

$$(\mathrm{MW_H} \cdot V_C) \times \frac{d[\mathrm{H^+}]_C}{dt} = \frac{dm_{\mathrm{H^+},C}}{dt}$$

Thus:

$$\begin{aligned}
\frac{dm_{\mathrm{H^+},C}}{dt} &= \mathrm{MW_H} \cdot V_C \cdot \mathrm{V_{max,H}} \cdot \frac{[\mathrm{HCO_3^-}]_c}{K_\mathrm{m} + [\mathrm{HCO_3^-}]_c} \\
&= \mathrm{MW_H} \cdot V_C \cdot \mathrm{V_{max,H}} \cdot \frac{m_{\mathrm{HCO_3^-},c}}{V_c \cdot \mathrm{MW_{HCO3}}} \cdot \frac{1}{K_\mathrm{m} + \frac{m_{\mathrm{HCO_3^-},c}}{V_c \cdot \mathrm{MW_{HCO3}}}} \\
&= \mathrm{MW_H} \cdot V_C \cdot \mathrm{V_{max,H}} \cdot \frac{m_{\mathrm{HCO_3^-},c}}{K_\mathrm{m} \cdot V_c \cdot \mathrm{MW_{HCO3}} + m_{\mathrm{HCO_3^-},c}}
\end{aligned}$$

Finally, to convert the H^+ mass into equivalent of HCO_3^- mass we multiply both sides by the molar mass ratio $\mathrm{MW_{HCO3}}/\mathrm{MW_H}$ and obtain:

$$\frac{dm_{HCO_3^-,c}}{dt} = \frac{v_{maxTHCO3} \cdot m_{HCO_3^-,c}}{K_{mTHCO3} \cdot V_c \cdot MW_{HCO3} + m_{HCO_3^-,c}}$$

where $v_{maxTHCO3} = MW_{HCO3} \cdot V_C \cdot V_{max,H}$ and $K_{mTHCO3} = K_m$. Using the previously estimated value of $V_{max,H}$, we find $v_{maxTHCO3} = 10.91 \cdot 10^{-3}$ pg s^{-1}. To obtain the maximum flux per surface unit we divide this value by the cell surface (we consider a cell radius $r_C = 6.55$ μm as in the previous section) so that, at the very end, $v_{maxTHCO3} = V_{maxTHCO3} \cdot S_C$ and $V_{maxTHCO3} = 2.024 \cdot 10^{-5}$ pg s^{-1} μm^{-2}.

It is known that the activity of bicarbonate transporters is regulated both by the intracellular and by the extracellular acidity (3, 7). To describe how bicarbonate fluxes depend upon pH_i we use the data in Fig.1, panel Biii, in ref. (7), while we use the data in Fig.2D in ref (3) to investigate how transport is affected by pH_e. The data were obtained by measuring proton fluxes in presence or in absence of the bicarbonate buffer. As explained in ref. (7), in the absence of the bicarbonate buffer only proton transporters are active, while in its presence both proton and bicarbonate transporters are active. The activity of THCO3 was then calculated by subtraction of these data.

As discussed in the main text we model this part with the following functions:

$$fpHi_{THCO3} = \frac{1}{2}\{1 + \tanh[\gamma_{THCO3} \cdot (pHi_{0,THCO3} - pH_i)]\} \quad (4)$$

$$fpHe_{THCO3} = \frac{1}{2}\{1 + \tanh[\lambda_{THCO3} \cdot (pH_e - pHe_{0,THCO3})]\} \quad (5)$$

We fit these nonlinear equations to experimental data and the results are shown in figures S4. Since experimental errors were not reported along with the original data the goodness-of-fit statistics cannot be computed for these fits. The fits returned the following best values for the parameters: $\gamma_{THCO3} = 4.2 \pm 0.72$, $pHi_{0,THCO3} = 6.9 \pm 0.02$, $\lambda_{THCO3} = 1.63 \pm 0.22$, $pHe_{0,THCO3} = 6.85 \pm 0.04$.

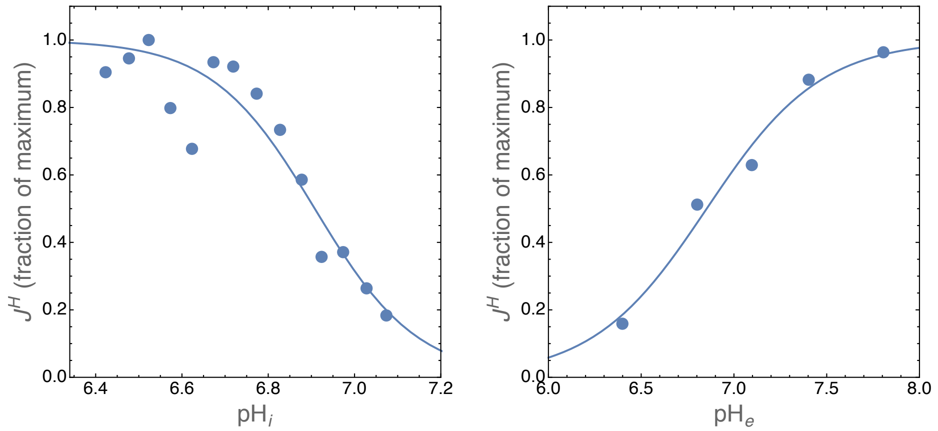

Figure S4: Activity of THCO3 transporters vs. extracellular and intracellular pH. Left panel: Symbols: data redrawn from ref. (7) and normalized with respect to the maximum observed value of J^H. Line: fit of experimental data with equation 4. Right panel: Symbols: data redrawn from ref. (3). The maximum flux J^H in this case was estimated by fit of raw data with a logistic equation. Line: fit of experimental data with equation 5.

The enzymatic activity of CA9

The enzyme CA9 follows the Michaelis-Menten kinetics. CA9 activity was measured by Li et al. (8) in experiments carried out with human breast cancer cells. They report the following values:

- initial CA9 concentration $[CA9]_0 = 1.3$ nM;
- kcat/Km $= 62 \pm 5$ μM^{-1} s^{-1};

- reaction volume = 2 ml
- cell density = $5 \cdot 10^5$ cells/ml.

Using these values we calculated the Michaelis-Menten parameter $V_{max} = 0.58$ mM s^{-1} per cell. This value was then converted to CO_2 mass units and divided by the cell surface assuming a cell radius of 6.55 μm to obtain the value listed in Table 1 of the main text. The data in Tab.1 in ref. (9) show that the K_m of the CA9 kinetics varies between 6.9 to 7.5 mM when measured in different experimental conditions, and we choose to take the average value of $K_m = 7.2$ mM.

CA9 expression in cells depends on the environmental oxygen concentration. Wykoff et al. (10) measured its expression in A549 cells (a human lung carcinoma cell line) grown in normoxic (i.e. 20% O_2) or hypoxic environments by western-blot. We measured the density of the bands in western-blot experiments shown in Fig.3B of their paper (10) using the open-source image processing software ImageJ (version: 2.00-rc-69/1.52r) and the results are shown in figure S5.

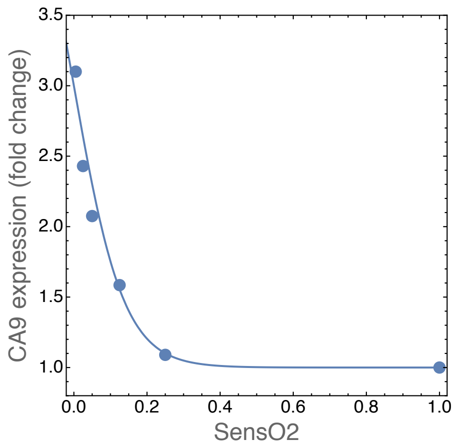

Figure S5: Expression of CA9 in cells grown under normoxic or hypoxic conditions. We used the software ImageJ to measure the density of the bands in western blots shown in Fig.3B in ref. (10). The results are expressed as fold-change expression with respect to CA9 protein band density observed for cells grown in a normoxic atmosphere (i.e. 20% O_2). The x-axis shows the fraction of oxygen to which the cells were exposed. This fraction corresponds to the parameter SensO2 in our model (see the main text). The data were fitted with equation 6 (line)

We fit the data with the following equation:

$$h_{CA9} = 3 + 2 \cdot \tanh\left(-\delta_{CA9} \cdot \text{SensO2}\right) \tag{6}$$

and obtain $\delta_{CA9} \simeq 7.3$.

REFERENCES

1. Chignola, R., and E. Milotti, 2005. A phenomenological approach to the simulation of metabolism and proliferation dynamics of large tumour cell populations. *Physical Biology* 2:8–22.

2. Chignola, R., A. Delfabbro, C. Dalla Pellegrina, and E. Milotti, 2007. *Ab initio* phenomenological simulation of the growth of large tumor cell populations. *Physical Biology* 4:114–133.

3. Hulikova, A., R. D. Vaughan-Jones, and P. Swietach, 2011. Dual role of CO2/HCO3- buffer in the regulation of intracellular pH of three-dimensional tumor growths. *Journal of Biological Chemistry* 286:13815–13826.

4. Luo, J., D. B. Kintner, G. E. Shull, and D. Sun, 2007. ERK1/2-p90RSK-mediated phosphorylation of Na+/H+ exchanger isoform 1 A role in ischemic neuronal death. *Journal of Biological Chemistry* 282:28274–28284.

5. Jandeleit-Dahm, K., K. M. Hannan, C. A. Farrelly, T. J. Allen, J. R. Rumble, R. E. Gilbert, M. E. Cooper, and P. J. Little, 2000. Diabetes-induced vascular hypertrophy is accompanied by activation of Na+-H+ exchange and prevented by Na+-H+ exchange inhibition. *Circulation Research* 87:1133–1140.

6. Boyer, M. J., and I. F. Tannock, 1992. Regulation of intracellular pH in tumor cell lines: influence of microenvironmental conditions. *Cancer Research* 52:4441–4447.

7. Hulikova, A., A. L. Harris, R. D. Vaughan-Jones, and P. Swietach, 2013. Regulation of intracellular pH in cancer cell lines under normoxia and hypoxia. *Journal of Cellular Physiology* 228:743–752.

8. Li, Y., C. Tu, H. Wang, D. N. Silverman, and S. C. Frost, 2011. Catalysis and pH control by membrane-associated carbonic anhydrase IX in MDA-MB-231 breast cancer cells. *Journal of Biological Chemistry* 286:15789–15796.

9. Hilvo, M., L. Baranauskiene, A. M. Salzano, A. Scaloni, D. Matulis, A. Innocenti, A. Scozzafava, S. M. Monti, A. Di Fiore, G. De Simone, et al., 2008. Biochemical characterization of CA IX: one of the most active carbonic anhydrase isozymes. *Journal of Biological Chemistry* 283:27799–27809.

10. Wykoff, C. C., N. J. Beasley, P. H. Watson, K. J. Turner, J. Pastorek, A. Sibtain, G. D. Wilson, H. Turley, K. L. Talks, P. H. Maxwell, et al., 2000. Hypoxia-inducible expression of tumor associated carbonic anhydrases. *Cancer Research* 60:7075–7083.

Appendix: C++ code

We list the code used to carry out simulations.

```
// Author: Nicola Piasentin
// Master Thesis Project
// The control of acidity in tumour cells: a biophysical model
// GSL libraries needed

#include <iostream>
#include <iomanip>
#include <fstream>
#include <stdlib.h>
#include <math.h>
#include <stdio.h>
#include <gsl/gsl_vector.h>
#include <gsl/gsl_multiroots.h>

using namespace std;

ofstream outData, outDatapH;

double m_CO2_C_old, m_H_C_old, m_HCO3_C_old, m_CO2_c_old, m_H_c_old, m_HCO3_c_old;
const double Pi = M_PI;

// sensors
const double SensO2 = 1.0;
const double SensATP = 1.0;

struct cell_params
{
  double MW_H;
  double MW_CO2;
  double MW_O2;
  double MW_HCO3;
  double MW_AcL;
  double r_C;
  double PM_CO2;
  double gAcL;
  double q_O2;
  double k1;
  double k2;
  double VMAXAcL;
  double K_mAcL;
  double a2cH_slope;
  double a2cH_thr;
  double c2aH_slope;
  double c2aH_thr;
  double VMAXNHE;
```

```
  double K_mNHE;
  double a;
  double l_NHE;
  double pH0_NHE;
  double VMAXTHCO3;
  double K_mTHCO3;
  double l_THCO3;
  double pHe0_THCO3;
  double g_THCO3;
  double pHi0_THCO3;
  double VMAXCA9;
  double K_mCA9;
  double d_CA9;
  double V_c;
  double dt;
};

int cell(const gsl_vector * x, void *params, gsl_vector * f)
{
  double MW_H = ((struct cell_params *) params)->MW_H;
  double MW_CO2 = ((struct cell_params *) params)->MW_CO2;
  double MW_O2 = ((struct cell_params *) params)->MW_O2;
  double MW_HCO3 = ((struct cell_params *) params)->MW_HCO3;
  double MW_AcL = ((struct cell_params *) params)->MW_AcL;
  double r_C = ((struct cell_params *) params)->r_C;
  double PM_CO2 = ((struct cell_params *) params)->PM_CO2;
  double gAcL = ((struct cell_params *) params)->gAcL;
  double q_O2 = ((struct cell_params *) params)->q_O2;
  double k1 = ((struct cell_params *) params)->k1;
  double k2 = ((struct cell_params *) params)->k2;
  double VMAXAcL = ((struct cell_params *) params)->VMAXAcL;
  double K_mAcL = ((struct cell_params *) params)->K_mAcL;
  double a2cH_slope = ((struct cell_params *) params)->a2cH_slope;
  double a2cH_thr = ((struct cell_params *) params)->a2cH_thr;
  double c2aH_slope = ((struct cell_params *) params)->c2aH_slope;
  double c2aH_thr = ((struct cell_params *) params)->c2aH_thr;
  double VMAXNHE = ((struct cell_params *) params)->VMAXNHE;
  double K_mNHE = ((struct cell_params *) params)->K_mNHE;
  double a = ((struct cell_params *) params)->a;
  double l_NHE = ((struct cell_params *) params)->l_NHE;
  double pH0_NHE = ((struct cell_params *) params)->pH0_NHE;
  double VMAXTHCO3 = ((struct cell_params *) params)->VMAXTHCO3;
  double K_mTHCO3 = ((struct cell_params *) params)->K_mTHCO3;
  double l_THCO3 = ((struct cell_params *) params)->l_THCO3;
  double pHe0_THCO3 = ((struct cell_params *) params)->pHe0_THCO3;
  double g_THCO3 = ((struct cell_params *) params)->g_THCO3;
  double pHi0_THCO3 = ((struct cell_params *) params)->pHi0_THCO3;
  double VMAXCA9 = ((struct cell_params *) params)->VMAXCA9;
  double K_mCA9 = ((struct cell_params *) params)->K_mCA9;
  double d_CA9 = ((struct cell_params *) params)->d_CA9;
  double V_c = ((struct cell_params *) params)->V_c;
  double dt = ((struct cell_params *) params)->dt;

  const double m_CO2_C = gsl_vector_get(x, 0);
  const double m_H_C = gsl_vector_get(x, 1);
  const double m_HCO3_C = gsl_vector_get(x, 2);
  const double m_H_c = gsl_vector_get(x, 3);
  const double m_HCO3_c = gsl_vector_get(x, 4);

  // intracellular carbon dioxide dynamics
  const double y0 = m_CO2_C - m_CO2_C_old - dt * (
    // internal rate
    SensO2 * q_O2 * MW_CO2 / MW_O2
    // chemical equilibrium
    - k1 * m_CO2_C + k2 * m_H_C * m_HCO3_C * 1000 * MW_CO2 / (4.0 / 3.0 * Pi * pow(r_C, 3.0) * MW_H *
    MW_HCO3)
    // diffusion
    + PM_CO2 * (m_CO2_c_old / V_c - m_CO2_C / (4.0 / 3.0 * Pi * pow(r_C, 3.0))) * (4.0 * Pi * pow(r_C,
    2.0))
```

```
  );

// intracellular hydrogen dynamics
const double y1 = m_H_C - m_H_C_old - dt * (
  // internal rate
  SensATP * gAcL * MW_H / MW_AcL
  // chemical equilibrium
  + k1 * m_CO2_C * MW_H / MW_CO2 - k2 * m_H_C * m_HCO3_C * 1000 / (4.0 / 3.0 * Pi * pow(r_C, 3.0) * MW_HCO3)
  // nu_MCT_in->out
  - (2.0 - tanh(c2aH_slope * (-log10(1000 * m_H_C / (4.0 / 3.0 * Pi * pow(r_C, 3.0) * MW_H))) - c2aH_thr)) * VMAXAcL * MW_H / MW_AcL
  * (4.0 * Pi * pow(r_C, 2.0)) * m_H_C / ((4.0 / 3.0 * Pi * pow(r_C, 3.0)) * K_mAcL * MW_H / MW_AcL + m_H_C)
  // nu_MCT_out->in
  + (2.0 - tanh(a2cH_slope * (-log10(1000 * m_H_c / (V_c * MW_H))) - a2cH_thr)) * VMAXAcL * MW_H / MW_AcL
  * (4.0 * Pi * pow(r_C, 2.0)) * m_H_c / (V_c * K_mAcL * MW_H / MW_AcL + m_H_c)
  // nu _NHE_in->out
  - SensO2 * SensATP * 0.5 * (1.0 + ((-log10(1000 * m_H_c / (V_c * MW_H))) - pH0_NHE) / (l_NHE + abs((-log10(1000 * m_H_c / (V_c * MW_H))) - pH0_NHE)))
  * VMAXNHE * (4.0 * Pi * pow(r_C, 2.0)) * pow(m_H_C, a) / (pow(4.0 / 3.0 * Pi * pow(r_C, 3.0) * MW_H * K_mNHE / 1000, a) + pow(m_H_C, a))
  );

// intracellular bicarbonate ions dynamics
const double y2 = m_HCO3_C - m_HCO3_C_old - dt * (
  // chemical equilibrium
  k1 * MW_HCO3 / MW_CO2 * m_CO2_C - k2 * m_H_C * m_HCO3_C * 1000 / (4.0 / 3.0 * Pi * pow(r_C, 3.0) * MW_H)
  // nu_THCO3_out->in
  + SensATP * (0.5) * (1.0 + tanh(l_THCO3 * (-log10(1000 * m_H_c / (V_c * MW_H)) - pHe0_THCO3)))
  * (0.5) * (1.0 + tanh(g_THCO3 * (pHi0_THCO3 - (-log10(1000 * m_H_C / (4.0 / 3.0 * Pi * pow(r_C, 3.0) * MW_H))))))
  * VMAXTHCO3 * (4.0 * Pi * pow(r_C, 2.0)) * m_HCO3_c / (V_c * K_mTHCO3 * MW_HCO3 / 1000 + m_HCO3_c)
  );

// extracellular hydrogen dynamics
const double y3 = m_H_c - m_H_c_old - dt * (
  // chemical equilibrium
  k1 * m_CO2_c_old * MW_H / MW_CO2 - k2 * m_H_c * m_HCO3_c * 1000 / (V_c * MW_HCO3)
  // nu_MCT_in->out
  + (2.0 - tanh(c2aH_slope * (-log10(1000 * m_H_C / (4.0 / 3.0 * Pi * pow(r_C, 3.0) * MW_H))) - c2aH_thr)) * VMAXAcL * MW_H / MW_AcL
  * (4.0 * Pi * pow(r_C, 2.0)) * m_H_C / ((4.0 / 3.0 * Pi * pow(r_C, 3.0)) * K_mAcL * MW_H / MW_AcL + m_H_C)
  // nu_MCT_out->in
  - (2.0 - tanh(a2cH_slope * (-log10(1000 * m_H_c / (V_c * MW_H))) - a2cH_thr)) * VMAXAcL * MW_H / MW_AcL
  * (4.0 * Pi * pow(r_C, 2.0)) * m_H_c / (V_c * K_mAcL * MW_H / MW_AcL + m_H_c)
  // nu _NHE_in->out
  + SensATP * SensO2 * 0.5 * (1.0 + ((-log10(1000 * m_H_c / (V_c * MW_H))) - pH0_NHE) / (l_NHE + abs((-log10(1000 * m_H_c / (V_c * MW_H))) - pH0_NHE)))
  * VMAXNHE * (4.0 * Pi * pow(r_C, 2.0)) * pow(m_H_C, a) / (pow(4.0 / 3.0 * Pi * pow(r_C, 3.0) * MW_H * K_mNHE / 1000, a) + pow(m_H_C, a))
  // nu_CA9
  + (3.0 + 2.0 * tanh(-d_CA9 * SensO2)) * VMAXCA9 * 4.0 * Pi * pow(r_C, 2.0) * m_CO2_c_old / (V_c * K_mCA9 * MW_CO2 / 1000 + m_CO2_c_old)
  * MW_H / MW_CO2
  );

// extracellular bicarbonate ions dynamics
const double y4 = m_HCO3_c - m_HCO3_c_old - dt * (
  // chemical equilibrium
  k1 * m_CO2_c_old * MW_HCO3 / MW_CO2 - k2 * m_H_c * m_HCO3_c * 1000 / (V_c * MW_H)
  // nu_THCO3_out->in
  - SensATP * (0.5) * (1.0 + tanh(l_THCO3 * (-log10(1000 * m_H_c / (V_c * MW_H)) - pHe0_THCO3)))
  * (0.5) * (1.0 + tanh(g_THCO3 * (pHi0_THCO3 - (-log10(1000 * m_H_C / (4.0 / 3.0 * Pi * pow(r_C, 3.0) * MW_H))))))
```

```
    * VMAXTHCO3 * (4.0 * Pi * pow(r_C, 2.0)) * m_HCO3_c / (V_c * K_mTHCO3 * MW_HCO3 / 1000 + m_HCO3_c)
    // nu_CA9
    + (3.0 + 2.0 * tanh(-d_CA9 * SensO2)) * VMAXCA9 * 4.0 * Pi * pow(r_C, 2.0) * m_CO2_c_old / (V_c *
    K_mCA9 * MW_CO2 / 1000 + m_CO2_c_old)
    * MW_HCO3 / MW_CO2
    );

  gsl_vector_set(f, 0, y0);
  gsl_vector_set(f, 1, y1);
  gsl_vector_set(f, 2, y2);
  gsl_vector_set(f, 3, y3);
  gsl_vector_set(f, 4, y4);

  return GSL_SUCCESS;
}

int main(void)
{
  const gsl_multiroot_fsolver_type *T;
  gsl_multiroot_fsolver *s;

  int status, time, j, k, perc;
  double dt, pH;// , pH_temp;
  size_t iter = 0;

  // respect units!
  const double MW_H = 1.0; // g/mol
  const double MW_CO2 = 44.0; // g/mol
  const double MW_O2 = 32.0; // g/mol
  const double MW_HCO3 = 61.0; // g/mol
  const double MW_AcL = 90.1; // g/mol
  const double r_C = 6.55; // mim
  const double PM_CO2 = 3.2 * pow(10, 4); // mim/s
  const double gAcL = 3.8 * pow(10, -4); // pg/s
  const double q_O2 = 3.5 * pow(10, -5); // pg/s
  const double k1 = 0.144; // 1/s
  const double k2 = 1.9 * pow(10, 5); // 1/(M*s)
  const double VMAXAcL = 9.58 * pow(10, -5); // pg/(s*mim^2)
  const double K_mAcL = 0.405 * pow(10, -3); // pg/mim^3
  const double a2cH_slope = 1.5; // adim
  const double a2cH_thr = 7.0; // adim
  const double c2aH_slope = 1.5; // adim
  const double c2aH_thr = 7.0; // adim
  const double VMAXNHE = 5.15 * pow(10, -7); // pg/(s*mim^2)
  const double K_mNHE = 0.196 * pow(10, -6); // pg/mim^3
  const double a = 2.67; // adim
  const double l_NHE = 0.076; // adim
  const double pH0_NHE = 7.1; // adim
  const double VMAXTHCO3 = 2.02 * pow(10, -5); // pg/(s*mim^2)
  const double K_mTHCO3 = 7.38 * pow(10, -3); // pg/mim^3
  const double l_THCO3 = 1.63; // adim
  const double pHe0_THCO3 = 6.85; // adim
  const double g_THCO3 = 4.2; // adim
  const double pHi0_THCO3 = 6.90; // adim
  const double VMAXCA9 = 9.47 * pow(10, -2); // pg/(s*mim^2)
  const double K_mCA9 = 7.2 * pow(10, -3); // pg/mim^3
  const double d_CA9 = 7.3; // adim
  const double V_c = 1.0 * pow(10, 12); // mim^3
  const double pKa = -log10(k1 / k2); // adim
  const double pH_cell = 7.40;  // adim

  const int max_iter = 1000; // max number of iterations for Newton-Raphson

  // input from the user
  cout << "Time interval: " << endl;
  cin >> dt;
  cout << "Total integration time: " << endl;
  cin >> time;
  cout << "Starting pH: " << endl;
```

```
cin >> pH;

// starting conditions
m_CO2_C_old = 5.39 * pow(10.0, -5) * (4.0 / 3.0 * Pi * pow(r_C, 3.0));
m_H_C_old = pow(10.0, -pH_cell - 3.0) * (4.0 / 3.0 * Pi * pow(r_C, 3.0));
m_HCO3_C_old = MW_HCO3 / MW_CO2 * m_CO2_C_old * pow(10.0, pH_cell - pKa);

m_CO2_c_old = 5.39 * pow(10.0, -5) * V_c;
m_H_c_old = pow(10.0, -pH - 3.0) * V_c;
m_HCO3_c_old = MW_HCO3 / MW_CO2 * m_CO2_c_old * pow(10.0, pH - pKa);

// friendly reminder
cout << endl;
cout << "**********************************************" << endl;
cout << endl;
cout << "Starting parameters" << endl;
cout << endl;
cout << "m_CO2_C  (pg): " << m_CO2_C_old << endl;
cout << "m_H_C    (pg): " << m_H_C_old << endl;
cout << "m_HCO3_C (pg): " << m_HCO3_C_old << endl;
cout << "m_CO2_c  (pg): " << m_CO2_c_old << endl;
cout << "m_H_c    (pg): " << m_H_c_old << endl;
cout << "m_HCO3_c (pg): " << m_HCO3_c_old << endl;
cout << "V_c   (mim^3): " << V_c << endl;
cout << "dt:            " << dt << endl;
cout << "steps:         " << time << endl;
cout << "time:          " << time * dt << endl;
cout << "starting pH:   " << pH << endl;
cout << "sensO2:        " << SensO2 << endl;
cout << "sensATP:       " << SensATP << endl;
cout << endl;
cout << "**********************************************" << endl;
cout << endl;
cout << "Running..." << endl;
cout << endl;

// FYI
perc = 10;
cout << "-- completed at: 0 %" << endl;

const size_t n = 5;
struct cell_params cell_p = { MW_H, MW_CO2, MW_O2, MW_HCO3, MW_AcL, r_C, PM_CO2, gAcL, q_O2, k1, k2,
  VMAXAcL, K_mAcL, a2cH_slope,
  a2cH_thr, c2aH_slope, c2aH_thr, VMAXNHE, K_mNHE, a, l_NHE, pH0_NHE, VMAXTHCO3, K_mTHCO3, l_THCO3,
  pHe0_THCO3, g_THCO3, pHi0_THCO3, VMAXCA9, K_mCA9, d_CA9, V_c, dt };

gsl_multiroot_function cell_f = { &cell, n, &cell_p };

double x_init[n] = { m_CO2_C_old, m_H_C_old, m_HCO3_C_old, m_H_c_old, m_HCO3_c_old }; // starting
  point
gsl_vector *x = gsl_vector_alloc(n);

for (k = 0; k < n; k++)
{
  gsl_vector_set(x, k, x_init[k]);
}

T = gsl_multiroot_fsolver_dnewton; // discrete Newton (discrete Jacobian)
s = gsl_multiroot_fsolver_alloc(T, n);

gsl_multiroot_function f = cell_f;

outData.open("cell_output.txt"); // output masses
outDatapH.open("cell_output_pH.txt"); // output pH

gsl_multiroot_fsolver_set(s, &f, x);

// output on masses file
outData << "# Starting parameters" << endl;
```

```
outData << endl;
outData << "# m_CO2_C   (pg): " << m_CO2_C_old << endl;
outData << "# m_H_C     (pg): " << m_H_C_old << endl;
outData << "# m_HCO3_C (pg): " << m_HCO3_C_old << endl;
outData << "# m_CO2_c   (pg): " << m_CO2_c_old << endl;
outData << "# m_H_c     (pg): " << m_H_c_old << endl;
outData << "# m_HCO3_c (pg): " << m_HCO3_c_old << endl;
outData << "# V_c    (mim^3): " << V_c << endl;
outData << "# dt:              " << dt << endl;
outData << "# steps:           " << time << endl;
outData << "# time:            " << time * dt << endl;
outData << "# starting pH:     " << pH << endl;
outData << "# sensO2:          " << SensO2 << endl;
outData << "# sensATP:         " << SensATP << endl;
outData << endl;
outData << "# step\tm_CO2_C\tm_H_C\tm_HCO3_C\tm_H_c\tm_HCO3_c" << endl;
outData << endl;

// output on pH file
outDatapH << "# Starting parameters" << endl;
outDatapH << endl;
outDatapH << "# m_CO2_C   (pg): " << m_CO2_C_old << endl;
outDatapH << "# m_H_C     (pg): " << m_H_C_old << endl;
outDatapH << "# m_HCO3_C (pg): " << m_HCO3_C_old << endl;
outDatapH << "# m_CO2_c   (pg): " << m_CO2_c_old << endl;
outDatapH << "# m_H_c     (pg): " << m_H_c_old << endl;
outDatapH << "# m_HCO3_c (pg): " << m_HCO3_c_old << endl;
outDatapH << "# V_c    (mim^3): " << V_c << endl;
outDatapH << "# dt:              " << dt << endl;
outDatapH << "# steps:           " << time << endl;
outDatapH << "# time:            " << time * dt << endl;
outDatapH << "# starting pH:     " << pH << endl;
outDatapH << "# sensO2:          " << SensO2 << endl;
outDatapH << "# sensATP:         " << SensATP << endl;
outDatapH << endl;
outDatapH << "# step\tpH_C\tpH_c\tpH_C HH\tpH_c HH" << endl;
outDatapH << endl;

// gonna need them
outData << fixed;
outData << setprecision(15);

outDatapH << fixed;
outDatapH << setprecision(15);

// first term
outData << "0" << "\t" << m_CO2_C_old << "\t" << m_H_C_old << "\t" << m_HCO3_C_old << "\t" <<
  m_H_c_old << "\t" << m_HCO3_c_old << endl;
outDatapH << "0" << "\t"
  << -log10(1000 * m_H_C_old / (4.0 / 3.0 * Pi * pow(r_C, 3.0))) << "\t"
  << -log10(1000 * m_H_c_old / V_c) << "\t" << log10((m_HCO3_C_old * MW_CO2 * k2) / (m_CO2_C_old *
  MW_HCO3 * k1))
  << "\t" << log10((m_HCO3_c_old * MW_CO2 * k2) / (m_CO2_c_old * MW_HCO3 * k1)) << endl;

// starts the time
for (j = 1; j < time; j++)
{
  iter = 0;

  do
  {
    iter++;

    status = gsl_multiroot_fsolver_iterate(s);

    if (status)   // check if solver is stuck
      break;

    status = gsl_multiroot_test_residual(s->f, 1e-6);
```

```
    } while (status == GSL_CONTINUE && iter < max_iter);

    // lazy...
    if (j * 100 / time == perc)
    {
      cout << "-- completed at: " << perc << " %" << endl;
      perc = perc + 10;
    }

    // output control
    if (j % 100 == 0)
    {

      outData << j * dt << "\t" << gsl_vector_get(s->x, 0) << "\t" << gsl_vector_get(s->x, 1) << "\t" << gsl_vector_get(s->x, 2)
        << "\t" << gsl_vector_get(s->x, 3) << "\t" << gsl_vector_get(s->x, 4) << endl;

      outDatapH << j * dt << "\t"
        << -log10(1000 * gsl_vector_get(s->x, 1) / (4.0 / 3.0 * Pi * pow(r_C, 3.0))) << "\t"
        << -log10(1000 * gsl_vector_get(s->x, 3) / V_c)
        << "\t" << log10((gsl_vector_get(s->x, 2) * MW_CO2 * k2) / (gsl_vector_get(s->x, 0) * MW_HCO3 * k1))
        << "\t" << log10((gsl_vector_get(s->x, 4) * MW_CO2 * k2) / (m_CO2_c_old * MW_HCO3 * k1)) << endl;

    }

    x_init[0] = gsl_vector_get(s->x, 0);
    x_init[1] = gsl_vector_get(s->x, 1);
    x_init[2] = gsl_vector_get(s->x, 2);
    x_init[3] = gsl_vector_get(s->x, 3);
    x_init[4] = gsl_vector_get(s->x, 4);

    m_CO2_C_old = x_init[0];
    m_H_C_old = x_init[1];
    m_HCO3_C_old = x_init[2];
    m_H_c_old = x_init[3];
    m_HCO3_c_old = x_init[4];

    // pH drop at time j*dt
    /*
    if (j == 200000)
    {
      m_H_C_old = m_H_C_old * pow(10.0, 1.0);
      pH_temp = -log10(m_H_C_old * 1000.0 / (4.0 / 3.0 * Pi * pow(r_C, 3.0) * MW_H));
      m_HCO3_C_old = MW_HCO3 / MW_CO2 * m_CO2_C_old * pow(10.0, pH_temp - pKa);
      x_init[1] = m_H_C_old;
      x_init[2] = m_HCO3_C_old;
    }
    */

    for (k = 0; k < n; k++)
    {
      gsl_vector_set(x, k, x_init[k]);
    }

    gsl_multiroot_fsolver_set(s, &f, x);

  }

  // closing
  cout << "-- completed at: " << perc << " %" << endl;
  cout << "-- done!" << endl;
  cout << endl;

  gsl_multiroot_fsolver_free(s);
  gsl_vector_free(x);
```

```
  outData.close();
  outDatapH.close();

  // because windows
  system("pause");

  return 0;

}
```
